# A greedy classifier optimization strategy to assess ion channel blocking activity and pro-arrhythmia in hiPSC-cardiomyocytes

**DOI:** 10.1371/journal.pcbi.1008203

**Published:** 2020-09-25

**Authors:** Fabien Raphel, Tessa De Korte, Damiano Lombardi, Stefan Braam, Jean-Frederic Gerbeau

**Affiliations:** 1 Inria, Paris, France; 2 NOTOCORD part of Instem, Le Pecq, France; 3 Ncardia, Leiden, Netherlands; US EPA, UNITED STATES

## Abstract

Novel studies conducting cardiac safety assessment using human-induced pluripotent stem cell-derived cardiomyocytes (hiPSC-CMs) are promising but might be limited by their specificity and predictivity. It is often challenging to correctly classify ion channel blockers or to sufficiently predict the risk for Torsade de Pointes (TdP). In this study, we developed a method combining *in vitro* and *in silico* experiments to improve machine learning approaches in delivering fast and reliable prediction of drug-induced ion-channel blockade and proarrhythmic behaviour. The algorithm is based on the construction of a dictionary and a greedy optimization, leading to the definition of optimal classifiers. Finally, we present a numerical tool that can accurately predict compound-induced pro-arrhythmic risk and involvement of sodium, calcium and potassium channels, based on hiPSC-CM field potential data.

## Author summary

**Fig 1:**
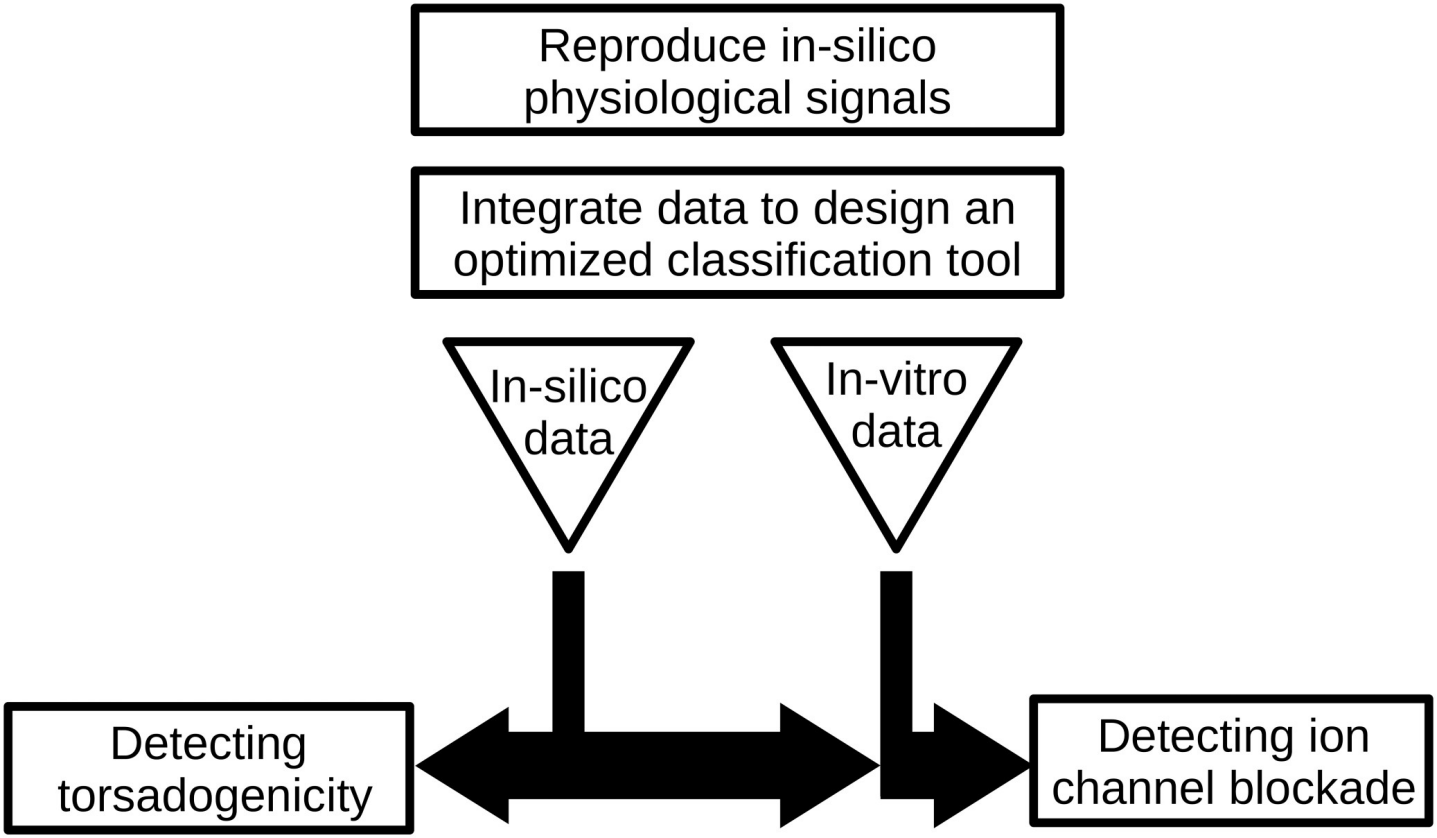
Summary of the main keypoints of the article.

Being able to measure the electrophysiology of human-induced pluripotent stem cell-derived cardiomyocytes (hiPSC-CMs) using multi-electrodes arrays (MEA) is promising in view of introducing novel drug screening methodologies in cardiac safety assessment in a preclinical setting. However, with new opportunities come new challenges. Data generated from hiPSC-CM MEA assays are challenging to interpret and translate to the clinical situation. Moreover, the observed experimental variability of the traces makes it difficult to assess whether we can systematically address classification problems such as channel blockade prediction, or arrhythmia risk. It would be of the utmost importance to understand if, in the Field potentials, there is enough information about these phenomena, and how it could be extracted. The method investigated is a first step towards this, and it is based on the construction of a dictionary of signal features: some of them are known markers used in electrophysiology, some are more agnostical signal characteristics. A goal oriented search is performed, in such a way that the input of the classifiers is found in order to maximise the success rate. Since, in general, the number of available experimental traces is not large enough to cover all the possible scenarios of interest, the experimental training set is complemented by an *in silico* training set. This method was applied to arrhythmic risk prediction on *in silico* data and channel blockade prediction on combined *in silico* and *in vitro* data. A conceptual scheme of the main points of the present contribution is presented in Fig 1.

## Introduction

The Comprehensive in vitro Proarrhythmia Assay (CiPA) is an initiative for a new paradigm in safety pharmacology to redefine the non-clinical evaluation of Torsade de Pointes (TdP) [1–3].

It aims to more precisely assess TdP risk *in vitro* by using a multifaceted approach that combines *in vitro* evaluations of electrophysiological responses in human-induced pluripotent stem cell-derived cardiomyocytes (hiPSC-CMs) and *in silico* models providing reconstructions of drug effects on ventricular electrical activity [4, 5].

Since CiPA, *in vitro* studies using hiPSC-CMs become an increasingly integrated part of today’s cardiac safety assessment. While encouraging, adequately predicting TdP risk of unknown drugs based on *in vitro* studies alone is challenging [6]. Besides, the analysis of the large data sets derived from those studies is often far from being automated.

One of the main challenges in proposing a high-throughput screening based on novel devices is often related to the variability of the signals measured, that could pose sensible questions about the ability to extract useful information from them. The main impact of the present work is related to this aspect, and the proposed framework can be considered as a first preliminary step towards the setup of a systematic procedure.

The main focus of the present study is to investigate a computational tool that combines statistical analysis and machine learning approaches (used in this context in [7]) to the mathematical modeling and the numerical simulations (*in silico* experiments) of the drug effects on the field potential (FP) of hiPSC-CMs obtained by multi-electrode array (MEA) technology.

Two problems of interest in the pharmacology community will be addressed: the first one is related to the prediction of the proarrhythmic behaviour of a drug, and the second one to the ion channels blockade (see Fig 1 for a summary of the main keypoints of the article). These are typical classification tasks. Some classification studies in cardiac electrophysiology were proposed in the literature, on simulated action potentials [7, 8] or ECG [9, 10].

The contributions of the present paper are the following:

1. A dictionary based greedy optimisation method is proposed, that selects the most pertinent signal features to maximise the classification score. This procedure helps correct the classical markers used to analyse Field Potential signals and provides encouraging results.
2. The *in vitro* dataset is complemented by an an *in silico* dataset. This makes it possible to explore all the possible scenarios and help mitigate the high-dimensional/low sample size regime potentially affecting the performances of the classifiers.
3. In constructing the signal database, the uncertainties affecting the experimental setup are accounted for. Despite many variability sources, the proposed approach aims at defining a robust classifier. Concerning the problems considered in the present manuscript, in the Field Potential there is enough information to provide an answer to them, irrespective of all the uncertainties affecting the experimental setup.
4. The proposed approach was tested on real data coming from actual experiments performed with MEA technology.

The work is structured as follows: the first part is dedicated to the methods used to reproduce *in silico* physiological signals (FP and calcium transient signals) based on the bidomain equations [11] and the O’Hara, Virág, Varró and Rudy (ORd) ionic model [12]. The relation between drug concentration and ion channel activity is rendered through scaling factors depending on IC50 values (as proposed in [13–15]. The outputs of the *in silico* model are the simulations of the Field Potentials (FPs) recorded from extracellular micro electrodes, and the averaged calcium transient on a well ([Ca^2+^]_i_).

The second part is dedicated to the description of the method used to integrate *in silico* experiments and *in vitro* data in order to design an optimised classification tool. The proposed approach is based on the construction of a dictionary of linear and non-linear forms applied to the set of *in vitro* and *in silico* data; a greedy algorithm is defined to build a sparse observation-to-prediction relation.

Finally, we applied the classification process in two situations: detecting torsadogenicity (TdP risk versus non-TdP risk) with a synthetic dataset and detecting ion channel blockade (for sodium, calcium or potassium channels) by the action of a given compound, on *in vitro* MEA data.

The classification results obtained show that the double greedy optimization strategy is effective in improving classifiers performances (with only a few parameters to be tuned) and is well adapted to study compound effects on hiPSC-CM electrophysiology that will aid in early and predictive cardiac safety assessment.

## Materials and methods

In this section, we present the method developed to improve the classification of electrophysiological regimes based on MEA signals. It consists in fusing together information coming from available experimental MEA data and numerical simulations in order to design the classifiers to be used.

**Fig 2:**
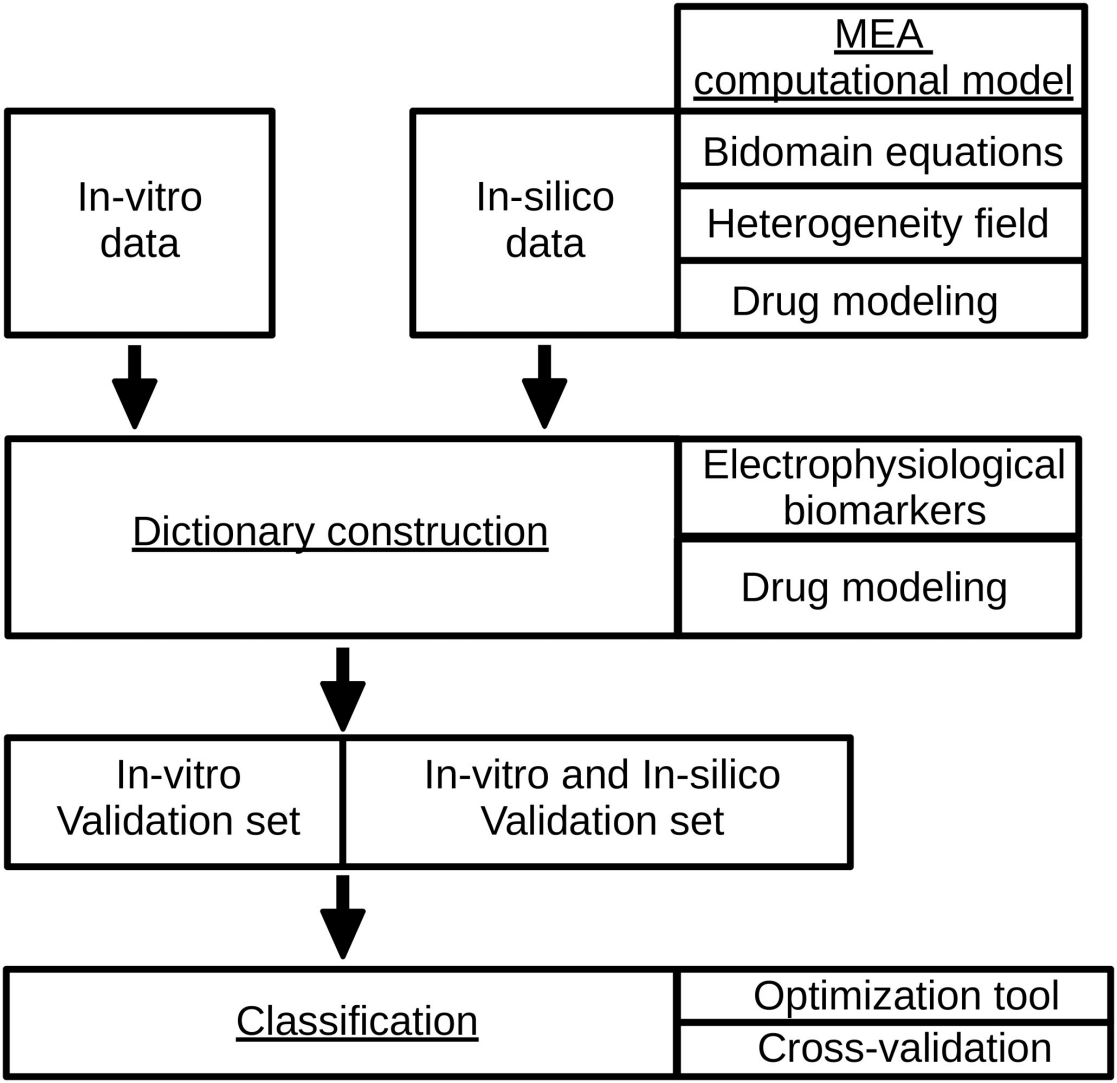
Scheme of the materials and methods section.

First, the experimental methods are described; then, we show the different models used to reproduce FPs and calcium signals (MEA computational model) and we end the section by presenting the optimised classification algorithm (Classification) and the definition of the dictionary entries (Dictionary construction). The structure of this section is shown in Fig 2.

### Experimental setup

The methods used to perform the experiments and acquire the recordings of the FPs are presented in detail below.

#### Cell culture

Human iPSC-CMs (Pluricyte Cardiomyocytes, Ncardia, Leiden, The Netherlands) were stored in liquid nitrogen until thawed and cultured onto 96 well MEA plates (Axion Biosystems, Inc., Atlanta, USA) according to manufacturer instructions. Briefly, the MEA plates were coated with fibronectin (50*μ*g/ml in PBS [+ Ca2+ & Mg2+], Sigma-Aldrich, St. Louis, MO, USA; Cat. No. F-1141) for 3 hours at 37°C and 5% CO2. After 3 hours of incubation time, the excess of fibronectin coating solution was removed and cells were plated in a 5 *μ*l droplet at a density of 25000 cells/well. After 1 hour of incubation (37°C and 5% CO2), 100*μ*l pre-warmed (37°C) medium (Pluricyte Cardiomyocyte Medium, Ncardia, Leiden, The Netherlands) was carefully added to each well. Cells were maintained in Pluricyte Cardiomyocyte Medium for 8 days and refreshments took place at day 1 post-thaw and subsequently every other day. MEA recordings were performed at day 8 post-thaw. The choice of these different parameters of the experimental setup was presented and commented in [16].

#### Test compounds

At day 8 post-thaw, medium was refreshed at least 2 hours before compound addition. The 12 test compounds were provided by the Chemotherapeutic Agents Repository of the National Cancer Institute and consisted of a random subset of CiPA compounds. The compounds were from 3 clinical TdP risk categories: low/no (Loratadine, Mexiletine, Diltiazem), intermediate (Clozapine, Chlorpromazine, Clarithromycine, Cisapride, Droperidol) and high (Ibutilide, Dofetilide, Bepridil, Azimilide) [17] (see Table 1). Chemical stock solutions at 1000 folds of the target concentrations were prepared under sterile conditions in DMSO and stored at −20°C, according to HESI Myocyte Phase II Validation Study Protocol instructions. The serial diluted compounds were further prepared in DMSO on the day of compound assay. The 10–fold final dilutions of the compounds were prepared with Pluricyte Cardiomyocyte Medium, for single time use only. Pluricyte Cardiomyocytes were exposed to four different concentrations of the compound, under sterile conditions in single point additions (i.e. one concentration per well) in five replicates for each concentration. Vehicle control was 0.1% DMSO.

**Table 1:** Section Channel classification: Experimental data information. IC50, Concentrations and *C*_*max*_ are given in *μM*. T/V column corresponds to the compound embedding into the Training set (T) or Validation set (V).

Compound*	IC50**			Concentration				*C*_*max*_ ***	T/V	Label
	hERG	Cav1.2	Nav1.5	#1	#2	#3	#4			
Loratadine	6.1	11.4	28.9	0.001	0.003	0.0095	0.03	0.00046	V	K [39], Ca [40]
Ibutilide	0.018	62.5	42.5	0.0001	0.001	0.01	0.1	0.1	T	K [41]
Droperidol	0.06	7.6	22.7	0.03169	0.10014	0.31646	1.0	0.02	T	K [41]
Mexiletine	62.2	125	38	0.1	1.0	10	100	2.5	T	Na [42], K [43]
Dofetilide	0.03	26.7	162.1	0.0003	0.001	0.0032	0.01	0.002	V	K [41]
Diltiazem	13.2	0.76	22.4	0.01	0.1	1.0	10	0.13	T	Ca [44]
Chlorpromazine	1.5	3.4	3.0	0.0951	0.3004	0.9494	3	0.0345	V	K [41], Ca [45], Na [46]
Clozapine	2.3	3.6	15.1	0.0951	0.3004	0.9494	3	0.07	T	K [47], Ca [40]
Clarithromycine	32.9	>30	NA	0.1	1	10	100	1.2	V	K [41]
Cisapride	0.02	11.8	337	0.0032	0.01	0.0316	0.1	0.0026	V	K [41]
Bepridil	0.16	1.0	2.3	0.01	0.1	1	10	0.03	V	K [41], Ca [48], Na [48]
Azimilide	<1†	17.8†	19†	0.01	0.1	1	10	0.07	V	K [49], Na [50], Ca [50]

^*^ TdP risk (low, medium or high) Colatsky *et al* [17].

^**^ From Ando *et al* [51].

^***^ From Blinova *et al* [6].

^†^ From Yao *et al* [50].

#### Ethic statement

All the experiments were performed under permits granted from the Commissie Medische Ethiek Leiden University Medical Center (permit number: NL45478.058.13).

#### MEA recordings

At day 8 post-thaw, 96 well MEA plates seeded with hiPSC-CMs were placed in the Maestro MEA device (768-channel amplifier) with an integrated heating system, temperature controller and data acquisition interface (Axion BioSystems, Inc., Atlanta, USA). The field potential traces of the hiPSC-CMs were recorded prior to (baseline) and 30 min after compound addition for 5 min. The recording conditions were at 37°C using Cardiac Standard filters and amplifiers in spontaneous cardiac mode (12.5 Hz sampling frequency, 2 kHz Kaiser Window, 0.1 Hz IIR). The beat detection threshold was 300 *μ*V.

### MEA computational model

This part provides a detailed description of the mathematical models used to simulate FPs in a realistic MEA geometry. Simulated FP studies were already performed for *in silico* assessment of drugs effects [14] or channel activity identification [18] and have shown the potency to reproduce and analyze compound effects on cardiac electrophysiology.

The first section concerns the bidomain equations, which governs the electrical activity propagation in a tissue. Since in a well the cells might not be perfectly uniformly distributed and the cell population might even be heterogeneous, a stochastic model of the population distribution was adopted, which is described in section Heterogeneity. In the last part (Drug modeling) we describe the compound simulation strategy, aiming at reproducing the experimental protocol used to classify reference compounds holding ion channel blocking properties.

#### Bidomain equations

To simulate MEA recordings, the bidomain Eq (1) were solved on a domain D, representing a well, by using a finite element method. The main geometrical hypothesis is that the cardiac cells in the well form a monolayer; therefore, to write the model, it is reasonable to consider a two dimensional domain, $D\subset R^{2}$.

The equations read:

$$\begin{array}{c} \left\{ \begin{array}{c} A_{m}C_{m}\frac{\partial V_{m}}{\partial t}+A_{m}I_{\text{ion}}\left(V_{m},w\right)-\nabla \cdot \left(\sigma_{i}\nabla V_{m}\right)-\nabla \cdot \left(\sigma_{i}\nabla \phi_{e}\right)=A_{m}I_{\text{app}},\\ -\nabla \cdot \left(\left(\sigma_{i}+\sigma_{e}\right)\nabla \phi_{e}\right)-\nabla \cdot \left(\sigma_{i}\nabla V_{m}\right)=\frac{1}{z_{\text{thick}}}\sum_{e_{k}}\frac{I_{\text{el}}^{k}}{|e_{k}|}\chi_{e_{k}}, \end{array}\right. \end{array}$$ (1)

where *A*_m_ is the surface area of the membrane per unit volume of tissue, *C*_m_ the membrane capacitance and *z*_thick_ the thickness of the cell layer. *V*_m_ and *ϕ*_*e*_ correspond respectively to the transmembrane potential and extracellular potential and *I*_ion_ is the ionic model depending on *V*_m_ and the gating variables. The propagation velocity is carried by the intracellular and extracellular conductivity (*σ*_*i*_ and *σ*_*e*_ respectively). The boundary conditions used with the bidomain equations are the following: *σ*_i_∇*ϕ*_i_ · **n** = 0 (with *ϕ*_*i*_ = *V*_*m*_ + *ϕ*_*e*_), and either *ϕ*_*e*_ = 0 on the region connected to the electrical ground or *σ*_e_∇*ϕ*_*e*_ · **n** = 0 elsewhere.

To take the impact of the electrodes on the signal into account, an imperfect electrode model [18] is coupled to the bidomain equations. The model is described in Eq 2.

$$\begin{array}{c} \frac{dI_{el}^{k}}{dt}+\frac{I_{el}^{k}}{\tau }=\frac{C_{el}}{\tau }\frac{\phi_{e,mean}^{k}}{dt}, \end{array}$$ (2)

where $\phi_{e,mean}^{k}$ is the averaged extracellular potential on the electrode *e*_*k*_, *R*_*el*_ and *C*_*el*_ are the electrode resistance and electrode capacitance respectively and *R*_*i*_ is the internal resistance of the measurement device. *τ* = *C*_*el*_(*R*_*i*_ + *R*_*el*_) is the time constant of the RC circuit. Then, the field potential $\phi_{f}^{k}$ measured on the electrode *e*_*k*_ is given by $\phi_{f}^{k}=R_{i}I_{el}^{k}$. For this study, electrodes parameters values used are summarized in Table A in S1 Text.

The ionic current *I*_ion_(*V*_*m*_, *w*) and the state variable *w* are provided by the ORd model [12]. Three types of cells are considered to mimic the monolayer heterogeneity (Heterogeneity): Epicardial, Mid-myocardial and Endocardial. These cell types are simulated through specific sets of parameters given in [12]. This model takes into account the main concentration dynamics ([Na^+^]_i_, [Ca^2+^]_i_ and [K^+^]_i_). The current *I*_app_ = *I*_app_(*x*, *y*, *t*) is the origin of the activation. The source is supposed to be located in a unique region and is defined as follows:

$$\begin{array}{c} I_{\text{app}}\left(x,y,t\right)=\{\begin{array}{c} I_{0}\exp[\frac{{\left(t-t_{0}\right)}^{2}}{2\sigma^{2}}]\;\text{if}\;{\left(x-x_{0}\right)}^{2}+{\left(y-y_{0}\right)}^{2}\leq r^{2},\\ \text{0,}\;\text{otherwise,} \end{array} \end{array}$$ (3)

where the position (*x*_0_, *y*_0_) is drawn randomly and *r* = 50*μm* is the radius of the source. *I*_0_ = −130*pA*/*pF* is the maximum stimulation value, *t*_0_ is the time when *I*_*app*_ is at its maximum and $\sigma =\frac{\Delta t}{6}$ with Δ*t* = 4ms.

The discretization of the partial differential equation was done in space using P1 Lagrangian finite elements and in time using backward differentiation formula (BDF) schemes with a time step of 0.1 ms. The ODE system governing the action potential modeling (*I*_*ion*_(*V*_*m*_, *ω*) in Eq 1) was solved using BDF scheme with adaptive time steps and order, whose implementation is provided by Sundials’ CVODE [19]. These space and time discretizations of the bidomain equations were already used in different studies (*in silico* ECG and *in silico* field potentials) and have shown qualitatively good results compared with real data [14, 18, 20–23].

To mimic experimental measurements, a 10*μV* standard deviation noise of a zero-mean Gaussian was added to FPs.

As some devices are able to get the intracellular calcium transient by fluorescence, we made the assumption that we have access to intracellular calcium transient data. We added a zero-mean Gaussian noise of 10^−3^ *μM* on the intracellular calcium transient obtained by simulation with the ORd model.

#### Heterogeneity

The hiPSC-CMs used in this study are > 70% pure cardiomyocytes based on positive Troponin T (TnT) expression. At least 70% of the TnT positive cells express a ventricular phenotype (based on ventricular myosin light chain 2 (MLC2v) expression and patch clamp technology). The other 30% of the cell population are of mesodermal origin. The actual distribution of these cells inside the well is unknown, and is a source of uncertainty that we need to take into account when developing the classifier in order to provide meaningful results in realistic applications.

To simulate this heterogeneity in the cell distribution inside a well, a space stochastic process was introduced, similarly to what was proposed in [24]: let (Z,A,P) be a complete probability space, *Z* being the set of outcomes, A a *σ*–algebra and P a probability measure:

$$\begin{array}{c} c(x,\zeta ):\Omega \times Z\to [0,1]. \end{array}$$ (4)

An hypothesis on the correlation of the process was made and expressed in Eq 5:

$$\begin{array}{c} f_{c}[(\begin{array}{c} x\\ y \end{array}),(\begin{array}{c} x^{\prime }\\ y^{\prime } \end{array})]=\text{exp}[-\frac{{\left(x-x^{\prime }\right)}^{2}+{\left(y-y^{\prime }\right)}^{2}}{2l_{c}^{2}}], \end{array}$$ (5)

that is, the correlation is normal and its length *l*_*c*_ was set to 0.25 mm, which corresponds approximately to the distance between two electrodes. A Karhunen-Loève expansion based on the diagonalisation of the correlation kernel was used in order to generate the heterogeneity fields (see [24] for details). As shown in [25] in a case of a low value for *l*_*c*_, the medium is homogenised which leads to a decrease of the repolarization phase. The parameter *l*_*c*_ = 0.25 mm was a good choice to qualitatively reproduce FP signals and more precisely the repolarization phase.

**Fig 3:**
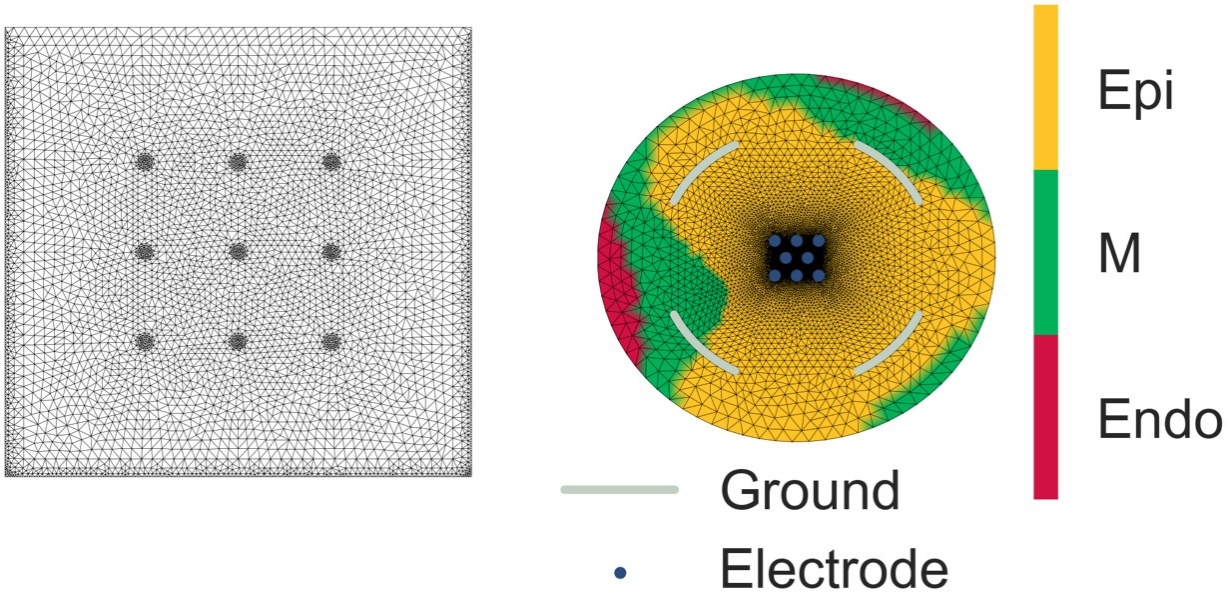
Section Heterogeneity: Finite element meshes of MEA used and example of heterogeneity field. Left: Finite element mesh representing one well including 9 electrodes of the 6–well MEA device from Multichannel Systems (used in TdP classification). MEA device documentation is available on: http://www.qichi-instruments.com/bookpic/20163120452599.pdf. Right: Finite element mesh representing one well including 8 electrodes of the 96–well MEA device from Axion Biosystems with an example of generated cell heterogneity field (used in Channel classification). MEA device documentation is available on: https://www.axionbiosystems.com/sites/default/files/resources/mea_plates-brochure-rev_06.pdf.

When discretised on the finite element space (P1 Lagrangian elements were used), a cell type was affected to each node of the finite element mesh according to the following rule:

$$\begin{array}{c} {\text{CellType}}_{i}=\{\begin{array}{c} \text{Epicardial},\;\text{if}\;c_{i}<\frac{1}{3}.\\ \text{Mid-myocardial,}\;\text{if}\;c_{i}>\frac{2}{3}.\\ \text{Endocardial,}\;\text{otherwise.} \end{array} \end{array}$$ (6)

where *c*_*i*_ ∈ [0, 1] is given by the random process *c* discretised at the node whose coordinates are **x**^(*i*)^ (see Eqs 4 and 5). Example of random heterogeneity obtained with this method is presented in Fig 3.

#### Drug modeling

**Fig 4:**
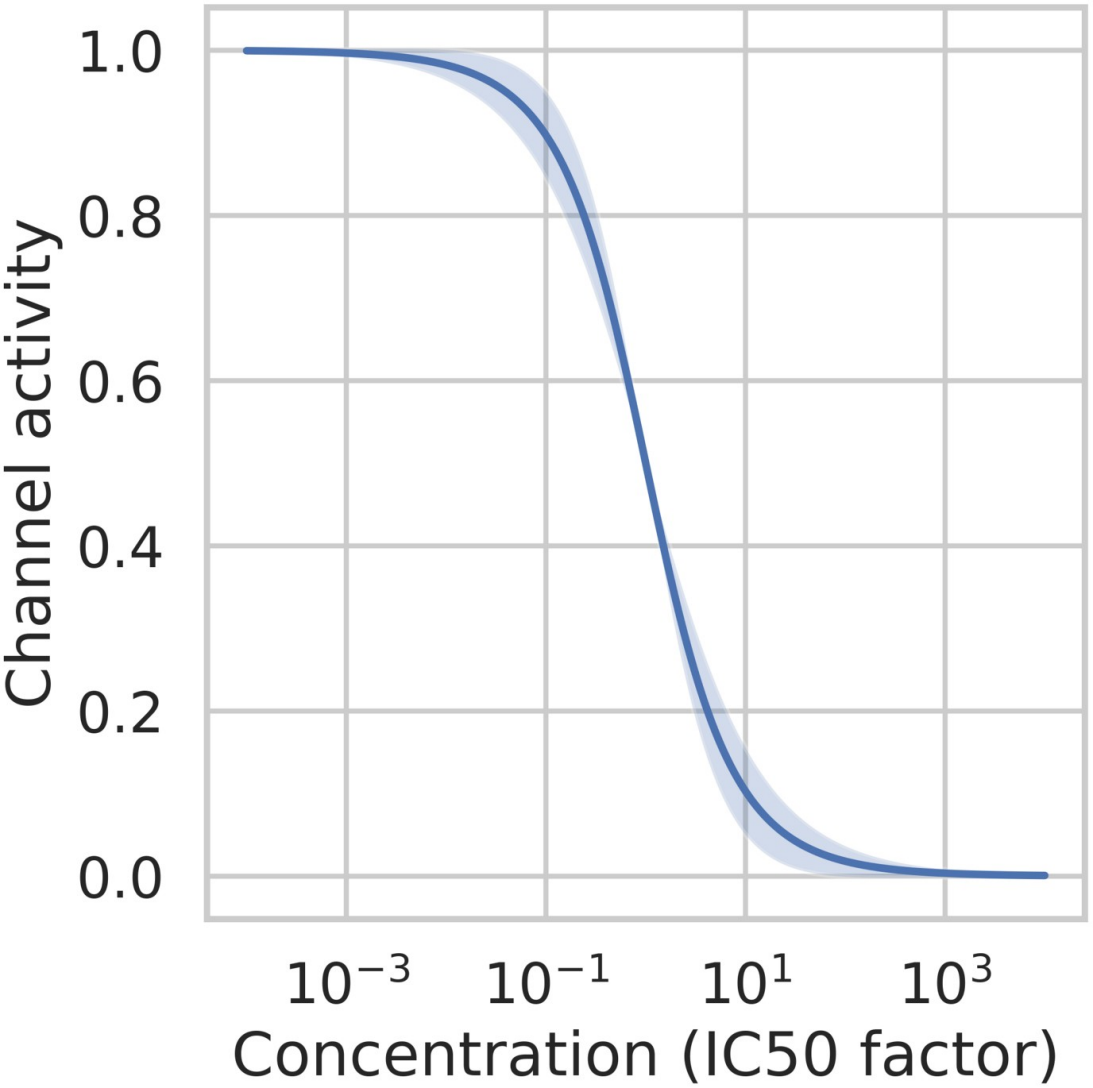
Section Drug modeling: Channel activity average and standard deviation for a Hill coefficient varying from 0.6 to 1.4. The abscisse is the concentration factor with respect to the *IC*50.

In this study we assume that a drug may affect only sodium, calcium and/or potassium channels. If the properties of a compound are known (e.g., IC50 for each ionic channel) then the conductance-block model [13–15] can be used to render its action on the ion channels. This model rewritten in Eq (7) qualitatively reproduces the expected compound effect on FPs [20].

$$\begin{array}{c} g_{s}=g_{control,s}[1+(\frac{\left[D\right]}{IC50_{s}}{)}^{n}\;{]}^{-1}, \end{array}$$ (7)

where *g*_*control*,*s*_ is the conductance of the channel s at control case (baseline), [*D*] is the concentration of the drug and *IC*50_*s*_ is the constant of the drug concentration at which current of channel s is blocked at 50%. We chose to set the Hill coefficient *n* at 1. The first reason is due to the confidence intervals of computed Hill coefficients for different compounds [26] which most of the time includes 1. The second reason comes from the use of the EFTPC in our simulations. Varying the Hill coefficient between 0.6 to 1.4, the standard deviation of the channel activity is lower than 0.05 for concentrations higher than the IC50 and lower than 0.03 for concentrations lower than the IC50 (see Fig 4). The use of the EFTPC leads to a low variability in the channel activity according to the Hill coefficient and studied compounds.

On the other hand, we can simulate unknown compounds, blocking randomly sodium, calcium and/or potassium channels.

**Fig 5:**
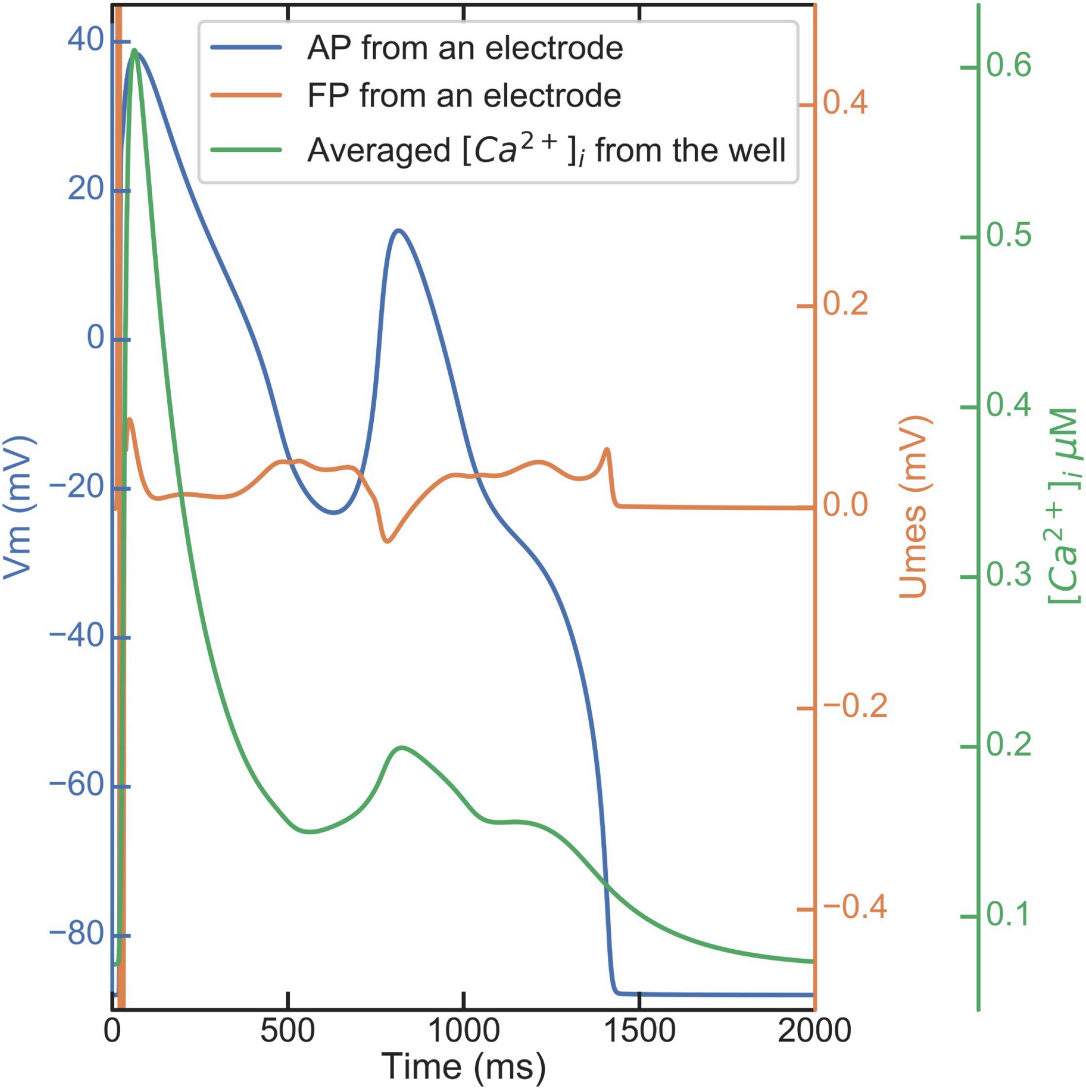
Section Drug modeling: EAD simulation. Transmembrane action potential (AP, blue), extracellular field potential (FP, orange) and intracellular calcium transient trace (green) in a simulated EAD case.

Fig 5 shows an example of simulated early afterdepolarization (EAD) as a result of blocking IKr current at 93.5%. The FP and intracellular calcium transient shapes are in good qualitative agreement with experimental signals [27, 28].

### Dictionary construction

In this section the details about the construction of the dictionary entries are provided and commented. As mentioned previously, the dictionary is a collection of linear and non-linear forms applied to the signals, corresponding to the definition of features (think, for instance, to the maximum of the signal, or its average, and so on). Since a greedy optimization strategy has been devised, and since the internal stages of the descent are easily parallelizable, the affordable dictionary size is potentially large (a few hundred in the study, potentially few thousands).

In the present work, the dictionary is divided into two parts:

- non-agnostic, or informed.
- agnostic

In the informed part we collect the biomarkers extracted from the signal, identified by the experts as correlated to some regime of interest. These quantities are meant to reveal a particular state of the system or alteration of a parameter. For instance, altering the sodium channel activity induces a modification in the depolarization amplitude [29]. The second part of the dictionary is agnostic, meaning that the linear and non-linear forms introduced are extracted from the signal as a mathematical object. The goal of the agnostic part of the dictionary is to enrich it, henceforth increasing the possibilities of computing from the dictionary an input leading to a good classification. The dictionary entries and their numbering are presented in Table D in S1 Text.

#### Dictionary entries: Electrophysiological biomarkers

**Fig 6:**
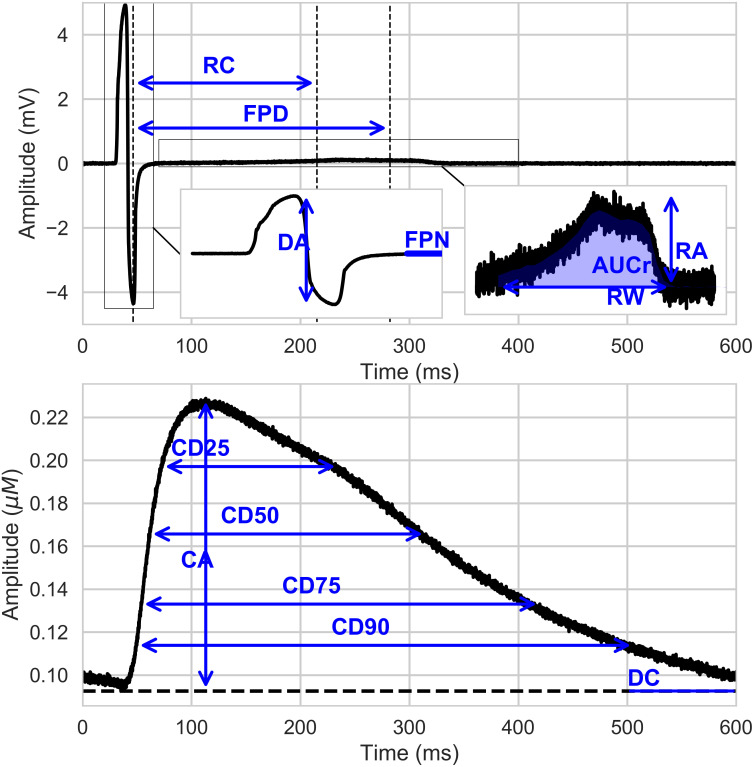
Section Dictionary entries: Electrophysiological biomarkers: List of the parameters computed on FP (up) and Calcium transient (down). RC: Repolarization Center; FPD: Field Potentiel Duration; DA: Depolarization Amplitude; FPN: Field Potential Notch; AUCr: Area Under Curve of the repolarizaztion wave; RA: Repolarization Amplitude; RW: Repolarization Width; CA: Calcium Amplitude; DC: ‘Drowsing Calcium’; CDX: Calcium Duration. See Sections Field Potential Biomarkers computation and Calcium Signals Biomarkers computation (S1 Text).

First, some intuitive biomarkers were extracted, *e.g*. depolarization amplitude (DA), field potential duration (FPD), etc. These quantities (called parameters in the electrophysiology community are presented in Fig 6.

**Remark**: In the electrophysiology community we often refer to parameters to designate quantities extracted from the experimental signals. In the present work, we follow the usage in applied mathematics and engineering communities, that name parameters the quantities affecting the state of the system and not the quantities read from the system observable.

Their computation follows the work in [24] and it is described in more details in Section Field Potential Biomarkers computation (S1 Text). Concerning the calcium transient signal computation, details are given in Section Calcium Signals Biomarkers computation (S1 Text).

**Fig 7:**
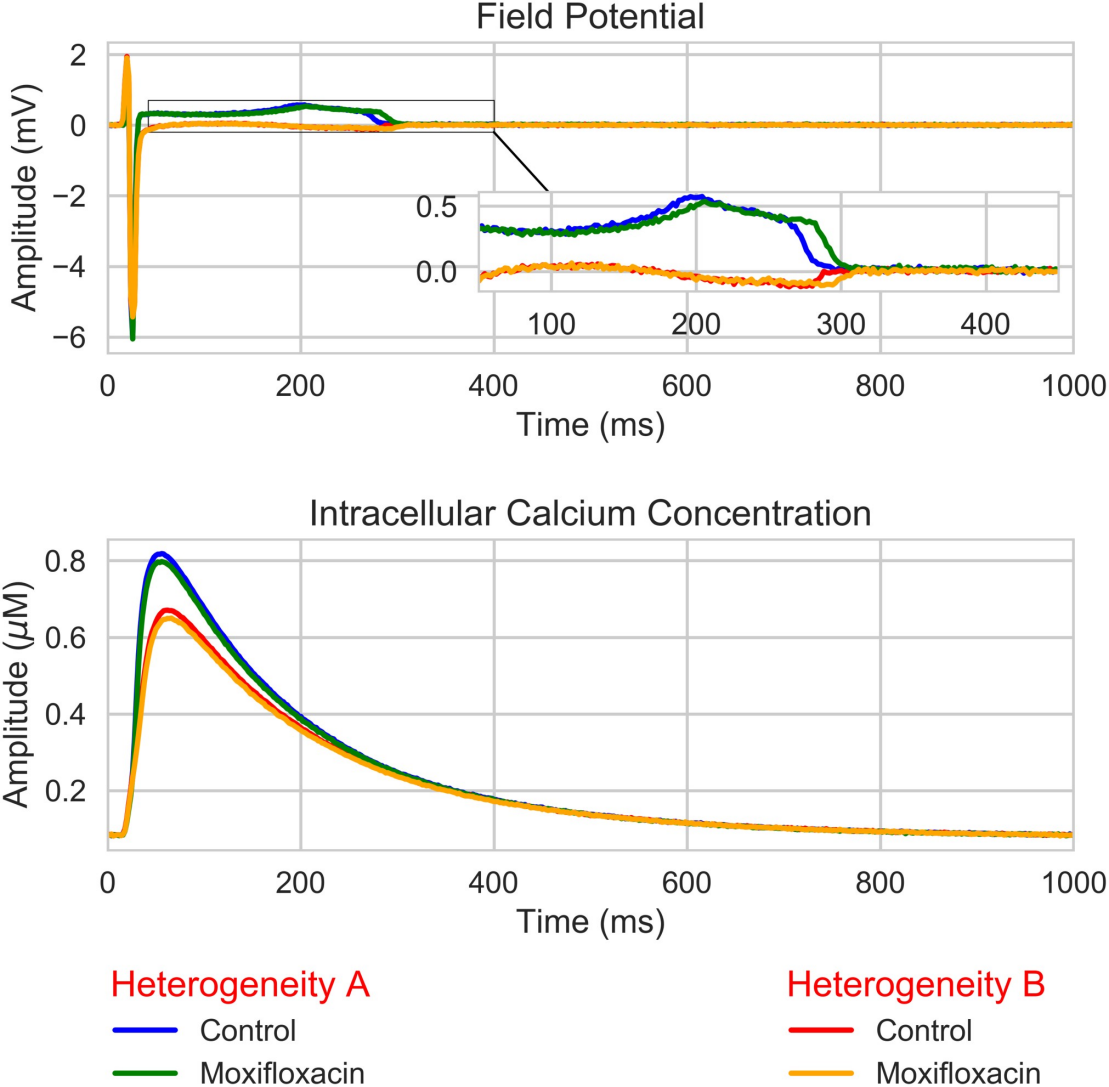
Section Dictionary entries: Electrophysiological biomarkers: Moxifloxacin simulation. Simulation of the effect of Moxifloxacin at effective free therapeutic plasma concentration (10.96*μM*, see Table F in S1 Text) on the FP (from one electrode) and intracellular calcium transient (from one well) for two different heterogeneity fields. A finite element mesh of 96-well MEA device from Axion Biosystems was used for this simulation (see right panel of Fig 3).

As these values of these biomarkers are computed in control and drug case, we decided to use relative values to the control case. For instance, the DA ratio is: $\frac{DA_{drug}}{DA_{ctrl}}$. The justification of this choice is shown in Fig 7. As we can see, even if the control case is different, the impact due to a compound is qualitatively the same regardless of the heterogeneity field.

**Fig 8:**
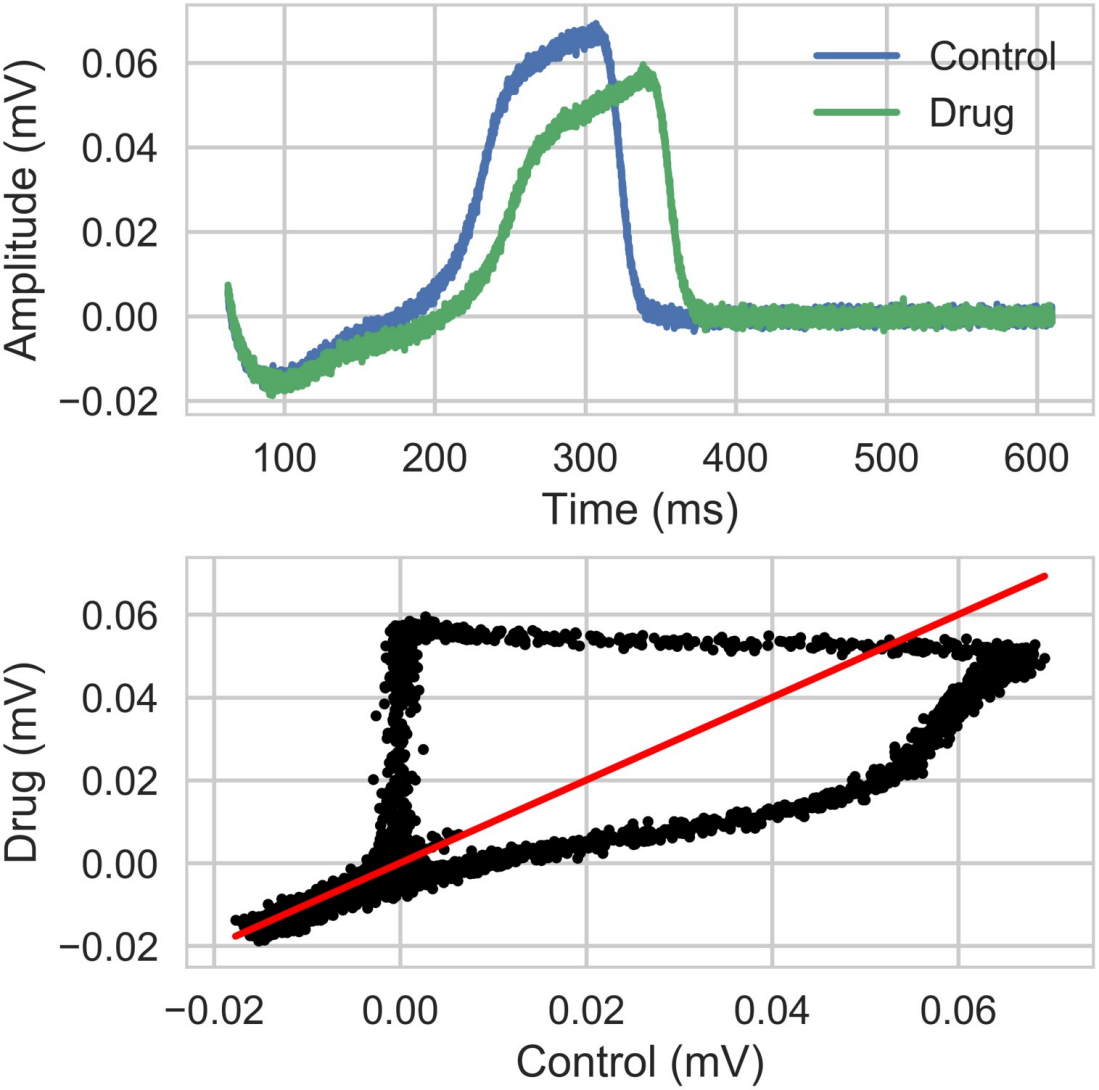
Section Dictionary entries: Electrophysiological biomarkers: Extended dictionary based on repolarization. Upper panel: FP repolarization. Lower panel: Repolarization of cells affected by a compound with respect to the control case repolarization. The red line corresponds to the case where the repolarization is not affected.

An example of an effect of a drug on the repolarization of the cells compared to baseline is presented in Fig 8.

In a case where a drug does not affect the repolarization, we should obtain a curve similar to *f*(*x*) = *x* (red line in Fig 8).

In the case where the repolarization is affected by a compound, a distortion appears on the signal (see black dots lower panel in Fig 8 corresponding to an increase in the FPD). Five markers (from K1 to K5) were extracted from the signal, with $FP_{rep}^{drug}$ and $FP_{rep}^{ctrl}$ the repolarization part of the FP for the drug and control case:

- Maximum distance: $max_{i}\sqrt{(FP_{rep}^{drug}\left(i\right)-FP_{rep}^{ctrl}\left(i\right){)}^{2}}$.
- *ℓ*^2^ norm: $\|FP_{rep}^{drug}-FP_{rep}^{ctrl}{\|}_{\ell^{2}}$.
- Average deviation: $\frac{1}{N}\sum_{i=1}^{N}(FP_{rep}^{drug}\left(i\right)-FP_{rep}^{ctrl}\left(i\right))$.
- Maximum deviation: $max_{i}(FP_{rep}^{drug}\left(i\right)-FP_{rep}^{ctrl}\left(i\right))$.
- Time of the maximum deviation.

#### Dictionary entries: Wavelets

**Fig 9:**
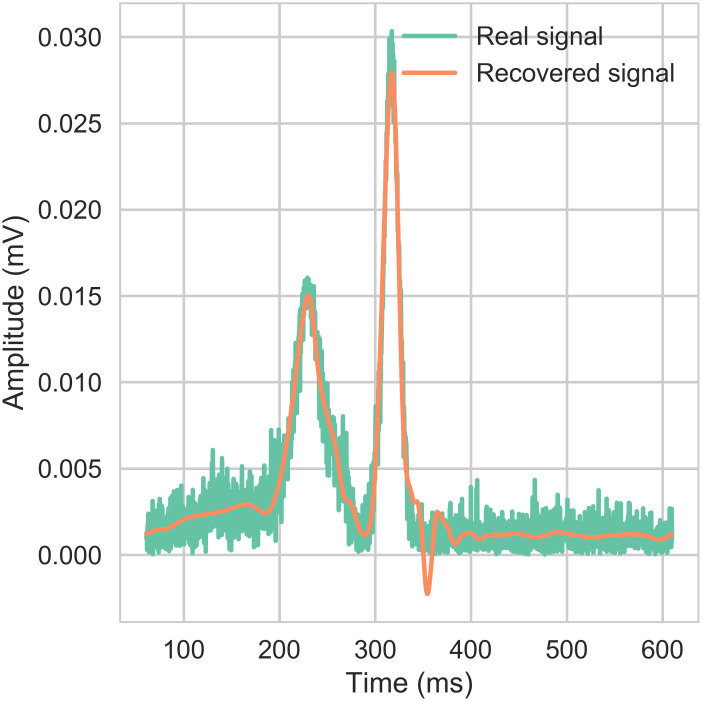
Dictionary entries: Wavelets: Signal reconstruction from wavelet coefficients. Reconstruction of the absolute difference between the drug and control signals for the plateau and repolarization phases, based on wavelets coefficients.

In order to construct the agnostic part of the signal, a wavelet decomposition was considered for the repolarization phase. The number of coefficients retained is such that the signal could be represented up to the noise level by the wavelets expansion. When a new signal is analyzed, only the selected coefficients (already computed for the training database) are then used to reconstruct the signal. If the *L*^2^ error is lower than an arbitrary value, we store these coefficients. Otherwise, we compute the new location and add the missing locations. The wavelet transform was done on the absolute difference between the drug case and the control case. An example of reconstruction is shown in Fig 9. The algorithm to get the positions is presented in the pseudo-code 1.

**Algorithm 1 Section Dictionary entries: wavelets: Wavelet coefficient**.

1: *N*_*s*_       ⊳ Number of signals to compute positions.

2: *thr*        ⊳ Threshold for the wavelets transform.

3: *v*_*p*_             ⊳ Empty array of positions.

4: **for** *i* ≔ 1 **to** *N*_*s*_ **do**

5:  *f*_*i*_               ⊳ Get the *i*^*th*^ signal.

6:  *c*_*wvlt*_ = *CWT*(*f*_*i*_, *thr*)   ⊳ Computes wavelets coefficients.

7:  $v_{p}^{nz}$      ⊳ Get the non-zeros positions of *c*_*wvlt*_.

8:  **if** i==1 **then**

9:   $v_{p}=v_{p}^{nz}$

10:  **else**

11:   $v_{p}=v_{p}⋃v_{p}^{nz}$

12:  **end if**

13: **end for**

### Classification

Given a molecule which is a candidate to become a drug, several questions arise concerning its impact on the electrical activity of cells. Basic questions like: *Is this drug blocking channel X?* or: *Is the drug potentially causing arrhythmia?* are naturally treated by solving a classification problem.

One of the main difficulties related to such a study is the curse of dimensionality [30] since we are dealing with high dimension, low sample size data. Otherwise stated, the function to be identified in view of setting up efficient classifiers is defined over a high dimensional domain and the number of available data is too low. To tackle this problem, numerical simulations were exploited. The rationale behind the strategy is twofold: first, we added virtual *in silico* experiments to the data set to increase the population size. Second, we exploited the simulations to extract meaningful low dimensional subsets of the data, which contributed to the mitigation of the high-dimensionality. Several methods are available in the literature to extract these low dimensional subsets [31].

However, the risk in using generic problem independent methods is that the subsets obtained could drastically reduce the amount of information conveyed about the quantity of interest to be classified.

Henceforth, we constructed a low dimensional subset of the data that has been exploited in the classifier construction, designed to deliver optimal classification performances. This method can be applied to all different classification techniques, and the result is classifier dependent.

#### Classification optimization

As stated, the aim was to set up a dimension reduction strategy in such a way that, when the extracted subset of the data is used as input for the classifier, we obtain optimal performances. The procedure reminds, in the spirit, the feature selection algorithms (and feature hashing), with some important differences, which will be highlighted later on.

First of all, for a classification problem, we needed to define a database of signal samples. Let $i\in N^{*},\;i\leq N_{s}$, the *i*-th signal sample is $z^{\left(i\right)}\left(t\right):R^{+}\to R^{m}$, where *m* depends on the number of electrodes of the device, as well as the ability for instance to read intracellular calcium transients. The database contains either real signals coming from MEA experiments or numerical signals.

After having collected a database of *N*_*s*_ signals, we defined a dictionary of linear and non-linear forms that has been applied to the signal. Let $B_{i}=\left(b_{1}^{i},\ldots b_{N_{b}}^{i}\right)$ be the dictionary entries (features extracted from signals) of the *i*^*th*^ sample:

$$\begin{array}{c} b_{j}^{\left(i\right)}\left(z^{\left(i\right)}\right):R^{m}\to R. \end{array}$$ (8)

The whole dictionary applied to the signals population is stored in a matrix $F_{s}\in R^{N_{s}\times N_{b}}$.

The input of the classifier is defined as a linear combination of the dictionary entries applied to a signal:

$$\begin{array}{c} x\in R^{d}, \end{array}$$ (9)

$$\begin{array}{c} x_{k}=\sum_{j=1}^{N_{b}}\omega_{kj}b_{j}\left(z\right),\;\;k=1,\ldots ,d. \end{array}$$ (10)

The weights *ω*_*kj*_ of the linear combination are stored in a matrix $\Omega \in R^{d\times N_{b}}$. The goal, is to find the weights matrix which defines the input space maximizing the classification score, for a given classification strategy. The problem is recast as a minimization of a cost function, describing the classification performances.

In the binary classification case, let *N*_*s*_ be the number of samples, *l*_*i*_ = {−1, 1} the true label of the *i*^*th*^ sample and $\hat{l_{i}}=\left\{-1,1\right\}$ the predicted label of the *i*^*th*^ sample associated with $\hat{p_{i}}\in \left[\frac{1}{2},1\right]$ the confidence of the classifier to be the predicted label. *n*_1_ (respectively *n*_−1_) is the number of samples labeled as 1 (respectively -1) and is introduced to avoid a possible bias due to unbalanced classes. Then, we have *n*_1_ + *n*_−1_ = *N*_*s*_. The *δ*_*i*_(*x*) function is the Dirac function (*δ*_*k*_(*x*) = 1 if *x* = *k*, 0 otherwise). Finally parameter *α* ≥ 1 is introduced to penalize the false positive case (*l*_*i*_ = 1 and $\hat{l_{i}}=-1$). The expression of the cost function is presented in Eq 11.

$$\begin{array}{c} s_{N_{s}}=-\frac{1}{N_{s}}\sum_{i=1}^{N_{s}}\hat{p_{i}}[\frac{N_{s}}{n_{1}}\delta_{1}\left(\hat{l_{i}}\right)\delta_{1}\left(l_{i}\right)+\frac{N_{s}}{n_{-1}}\delta_{-1}\left(\hat{l_{i}}\right)\delta_{-1}\left(l_{i}\right)-\alpha \frac{N_{s}}{n_{1}}\delta_{-1}\left(\hat{l_{i}}\right)\delta_{1}\left(l_{i}\right)-\frac{N_{s}}{n_{-1}}\delta_{1}\left(\hat{l_{i}}\right)\delta_{-1}\left(l_{i}\right)]. \end{array}$$ (11)

With this formulation, the minimum value of $s_{N_{s}}$ is -2 and the highest value is 1 + *α*. See the demonstration in Cost function demonstration: 2 classes (S1 Text). The rationale of including the terms ${\hat{p}}_{i}$ in the cost function is to better describe the performances of the classifier, accounting for the confidence in the classification, and not merely on the success rate. This aims at setting up a robust classification tool.

Following the same principles, the cost function can be extended to *k* classes as shown in Eq 12:

sNs=-1Ns∑i=1Nspi^{∑j=1k[Nsnjδj(li^)δj(li)-Nsnjαj∑m=1m≠jkδm(li^)δj(li)]}, (12)

where *n*_*j*_ is the number of samples labeled *j* and *α*_*j*_ > 0 the weight assigned if the predicted label is not the class *j*. The value obtained for the best case is −*k* whereas the cost obtained in the worst case is $\sum_{j=1}^{k}\alpha_{j}$. Demonstrations are presented in Cost function demonstration: general case (S1 Text). In the case where we do not penalize classes, all the *α*_*j*_ are equal to 1.

A regularization term was added to the cost function:

$$\begin{array}{c} s_{N_{s},reg}=s_{N_{s}}+\beta (\sum_{k=1}^{d}\left(1-\sum_{i=1}^{N_{b}}\omega_{ki}^{2}\right)), \end{array}$$ (13)

where $\beta \in R^{+}$ is a penalization parameter. This term aims at breaking the scaling invariance of the linear combination of the dictionary entries. In particular, if a linear classifier is used, let α∈R,α≠0, the classification score when using *α* **Ω** is the same as **Ω**, irrespective of the value of *α*.

The optimization problem reads:

$$\begin{array}{c} \Omega^{*}=\text{arg}\underset{\Omega \in R^{d\times N_{b}}}{\text{inf}}s_{N_{s},reg}. \end{array}$$ (14)

The optimization problem is challenging to be solved for several reasons: first, the dimension *d* of the input space to be found is not known *a priori*, but has to be determined. Second, given realistic signals, the number of features that can be extracted, and hence the size of the dictionary, can be quite large, leading to an optimization problem on a large dimensional space. To mitigate these difficulties, a greedy optimization strategy was adopted. The rationale is the following: we are interested in finding an input space of small dimension (to overcome the curse of dimensionality), and it is preferable that each input dimension is expressed as a sparse linear combination of the dictionary entries, to avoid overfitting problems. Henceforth, we start by defining an input space of dimension *d* = 1. For this, the weight matrix reduces to a vector. Assuming that only one dictionary entry can be used, an optimization is performed to choose the entry. Then, we look for a combination of two dictionary entries, and so on, till the score variation when adding an entry is less than a prescribed tolerance. When the first component of the input space *x*_1_ is determined, we look for possible improvements by setting *d* = 2 and computing *x*_2_ in the same way, when *x*_1_ is the one found at the previous step of the algorithm. The greedy descent stops when the variation of the classification score is lower than an arbitrary tolerance. This double greedy strategy is defined in the pseudo-code Algorithm 2. The inner steps of the algorithm require the solution of a small size optimization problem, which is carried out by using a stochastic evolutionary strategy CMA-ES (presented in [32]).

**Algorithm 2 Section Classification optimization: Sparse optimised classification**.

1: *F*_*s*_: dictionary matrix        ⊳ $F_{s}\in R^{N_{s}\times N_{b}}$.

2: *l*: output vector        ⊳ Vector of size *N*_*s*_.

3: **Ω** = 0        ⊳ Initialize matrix of weights $\Omega \in R^{d\times N_{b}}$ with 0 values.

4: **for** *id* = 1 **to** *d* **do**  ⊳ Loop on the number of desired feature space dimension.

5:  *V*_*pos*_ = (1, 2, …, *N*_*b*_)     ⊳ Initialize the vector of the entries positions.

6:  *N*_*pos*_ = #(*V*_*pos*_)      ⊳ Length of *V*_*pos*_, number of available positions.

7:  *ω*_*id*_ = 0      ⊳ Vector of size *N*_*b*_. Weights of the *id*^*th*^ dimension.

8:  **for** *ic* = 1 **to** *N*_*comp*_ **do**   ⊳ Loop on the desired number of components to take into account for dimension *id*.

9:   *p*_*save*_, *ω*_*save*_    ⊳ Empty vectors. Store respectively best components position and best weights.

10:   Ω_*save*_ = 0    ⊳ Empty matrix. Store best weights for all tested components. $\Omega_{save}\in R^{ic\times N_{pos}}$.

11:   *s*_*pos*_ = 0       ⊳ Vector with length of *N*_*pos*_. Store computed cost.

12:   **for** *ip* = 1 **to** *N*_*pos*_ **do**   ⊳ Loop on the number of available positions.

13:    *ω* = 0         ⊳ Initialize weight vector of size *N*_*b*_.

14:    *ω*[*p*_*save*_] = *ω*_*save*_     ⊳ Initialize first components (if *ic* > 1).

15:    *ω*[*V*_*pos*_[*ip*]] = 1      ⊳ Initialize the tested component.

16:    **Ω**[*id*,:] = *ω*   ⊳ Initialize the *id*^*th*^ dimension of the weights matrix.

17:    **while** isConverged==False **do**

18:     *M* = *F*_*s*_.**Ω**^⊤^[:,: *id*]    ⊳ Classification input matrix $M\in R^{N_{s}\times id}$.

19:     $s_{N_{s}}=\text{classification}\left(M,l\right)$     ⊳ Cost computation.

20:     *isConverged* = checkConvergence    ⊳ Number of iterations, stagnation,…

21:     *ω*[[*p*_*save*_, *V*_*pos*_[*ip*]]] = *ω*_*new*_    ⊳ CMA-ES. #(*ω*_*new*_) = *ic*.

22:     **Ω**[*id*,:] = *ω*            ⊳ Store weights.

23:     $s_{pos}\left[ip\right]=s_{N_{s}}$      ⊳ Store the computed cost.

24:     Ω_*save*_[:, *ip*] = *ω*_*new*_

25:    **end while**

26:   **end for**

27:   *p*_*min*_ = *argmin*(*s*_*pos*_[*ip*])      ⊳ Get the position of the best entry.

28:   *p*_*save*_ = (*p*_*save*_, *V*_*pos*_[*p*_*min*_])      ⊳ Concatenate the position.

29:   *ω*_*save*_ = Ω_*save*_[:, *p*_*min*_]         ⊳ Get the best weights.

30:   *V*_*pos*_ = *V*_*pos*_∖*V*_*pos*_[*p*_*min*_]        ⊳ Remove the position.

31:   *N*_*pos*_ = #(*V*_*pos*_)          ⊳ (= *N*_*pos*_ − 1). Length of *V*_*pos*_.

32:   *ω*_*id*_[*p*_*save*_] = *ω*_*save*_

33:  **end for**

34:  **Ω**[*id*,:] = *ω*_*id*_     ⊳ Actualize the weights for the *id*^*th*^ dimension.

35: **end for**

The stopping criterion can be derived based on the analysis of the cost function. We propose to find the minimum variation which ensures that the classification does not change. It means that only the probabilities can differ between two consecutive cost computations without changing the predicted class. This corresponds to an increase of the margin between the classes. More details are given in Stop criterion based on the success rate variation (S1 Text).

**Fig 10:**
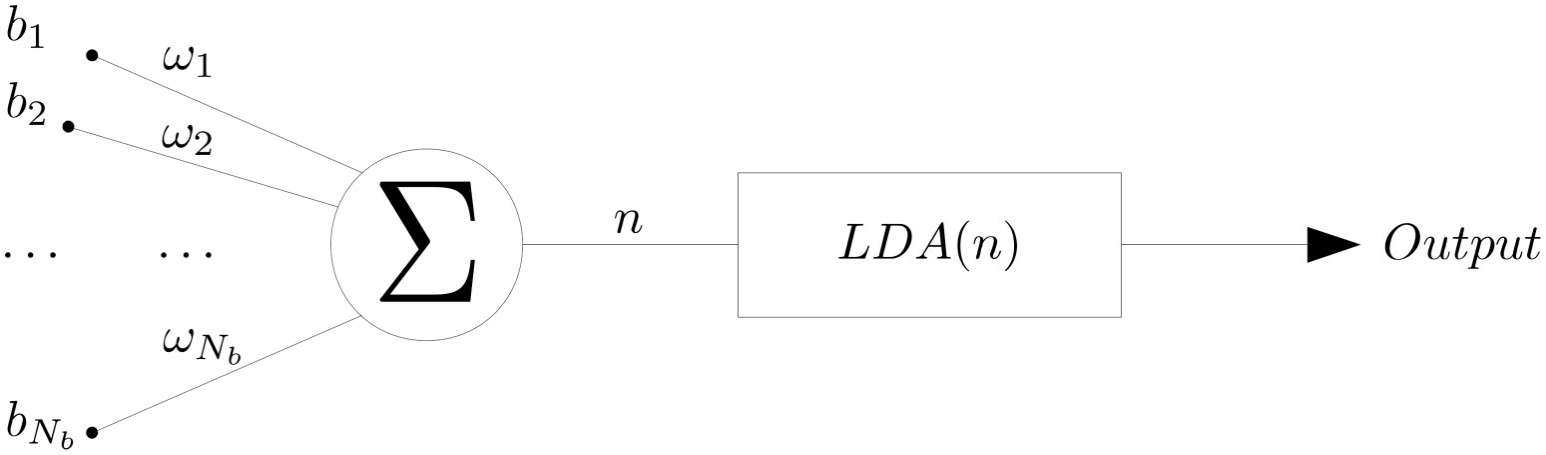
Section Classification optimization: Optimised algorithm in one dimension interpreted as a neural network.

**Fig 11:**
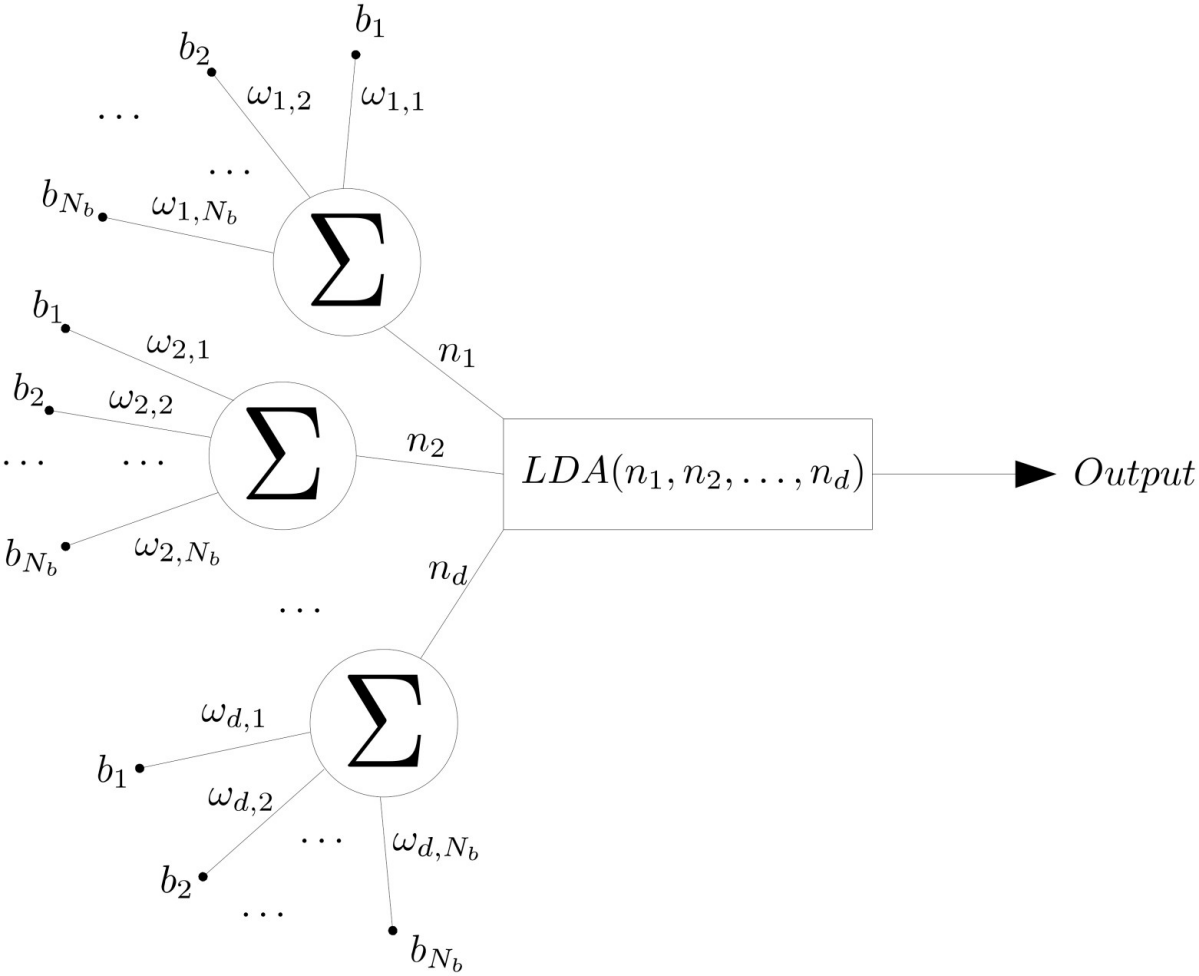
Section Classification optimization: Optimised algorithm for d dimensions interpreted as a neural network.

**Remark**: the presented algorithm can be interpreted as a simple perceptron [33] with one hidden layer as shown in Fig 10 where all the weights are equal to zero except those selected by the algorithm. Instead of using usual activation function as sigmoid or Heaviside [34] we used the linear discriminant analysis (LDA) technique [35]. The analogy to the back propagation to compute the weights corresponds here to the CMA-ES algorithm. For a given set of weights, the obtained output is compared to the real solution through the cost function. Then a correction is made on the weight until convergence. Fig 11 shows the multidimensional case where the LDA classifier takes the different linear combinations computed for each dimension as input.

#### Cross-Validation

As inputs are computed to maximize the classification, a risk is to lose the generalization capacity of a good classifier. To prevent the overfitting and to increase the robustness of the strategy a random k-fold cross-validation was used. A stratification was applied on the data to ensure the conservation of the output repartition in each fold.

The pseudo-code is described in Algorithm 3 and the corresponding Scikit-Learn method was used.

**Algorithm 3 Section Cross-Validation: Randomized K-fold cross-validation procedure**.

1: *M*: input matrix

2: *l*: output vector

3: *N*_*fold*_ = 2       ⊳ Number of folders for each K-Fold.

4: *N*_*kfold*_ = 500         ⊳ Number of K-Fold.

5: *E* = (1, 2, …, *N*_*s*_)       ⊳ Sample numbering.

6: *Cnt* = 0       ⊳ Initialize counter vector of size *N*_*s*_.

7: *P* = 0      ⊳ Initialize matrix $P\in R^{N_{s}\times 2}$ with 0 values.

8: **for** *i* ≔ 1 **to** *N*_*kfold*_ **do**

9:  *E*′ = *getFolders*(*E*, *N*_*fold*_, *l*)   ⊳ Generate the *N*_*fold*_ folders with respect to the stratification.

10:  **for** *j* ≔ 1 **to** *N*_*fold*_ **do**

11:   *pos*_*test*_ = *E*′[*j*]         ⊳ Testing folder (vector of indices).

12:   *pos*_*train*_ = *E*∖*pos*_*test*_        ⊳ Complementary of *pos*_*test*_.

13:   *M*_*train*_ = *M*[*pos*_*train*_,:]        ⊳ Extract train submatrice.

14:   *l*_*train*_ = *l*[*pos*_*train*_]         ⊳ Extract train subvector.

15:   *M*_*test*_ = *M*[*pos*_*test*_,:]        ⊳ Extract test submatrice.

16:   *l*_*test*_ = *l*[*pos*_*test*_]          ⊳ Extract test subvector.

17:   *clf* = Train on (*M*_*train*_, *l*_*train*_)      ⊳ Train the classifier.

18:   *proba* = Test on (*clf*, *M*_*test*_)   ⊳ Test the new data with the classifier.

19:   *P*[*pos*_*test*_,:] = *P*[*pos*_*test*_,:] + *proba*    ⊳ Add the new probabilities.

20:   *Cnt*[*pos*_*test*_] = *Cnt*[*pos*_*test*_] + 1

21:  **end for**

22: **end for**

23: **for** *i* ≔ 1 **to** *N*_*s*_ **do**

24:  *P*[*i*,:] = *P*[*i*,:]/*Cnt*[*i*]    ⊳ Compute averaged probability for the *i*^*th*^ sample.

25: **end for**

The repetition of the random K-fold strategy allows the convergence of the weights regardless of the training and test set generated. The higher the number of weights to determine, the higher should be the number of random K-fold.

## Results

Two different studies were performed in the present work: classify compounds for their risk on TdP; classify compounds for their ion channel blocking properties.

The results of these are presented hereafter.

In the first part of section TdP classification, we describe the study results based on the conductance-block model (see Eq 7). Using this model, we classified the TdP risk of 86 known compounds based on simulated data using the compound’s IC50 values for blocking sodium, potassium and calcium currents and the effective free therapeutic plasma concentration (EFTPC) values, reported by the literature.

In the second study (section Channel classification) we classified compounds based on experimental data. The outcome consists in identifying which channel is affected (sodium, calcium or potassium) by a compound. These experiments were performed for 12 compounds using Pluricyte Cardiomyocytes. Five of them were used for the training set and seven of them for the validation. Because of the low sample size of data, a simulated database was generated to enrich the training set.

The stop criterion used for the following results is when the cost variation between the last two components is lower than 5%. The penalization parameter *β* described in Eq 13 was set to 0.1.

### TdP classification

This section is dedicated to the torsadogenicity risk classification. Only simulated data are considered for this study. To predict the risk of TdP of a wide range of compounds, we simulated the application of 86 known compounds previously reported by [7].

#### Tests setup

The numerical choices leading to the results are summarised hereafter:

- The false positive part in the cost function (see Eq 11) was taken *α* = 2, to minimise the false positive rate.
- Bidomain equation parameters are summarized in Table B in S1 Text.
- Drugs were modeled using Eq 7 presented in section Drug modeling. The IC50 values for each compound are given in [13, 36]. Concentrations chosen to simulate compounds are the effective free therapeutic plasma concentrations (EFTPC). These values are listed in Tables E and F in S1 Text. Eighteen drugs were modeled twice because of their different IC50 and EFTPC observed in the literature (see Tables E and F in S1 Text).
- The corresponding channels blocked in the ORd model are *I*_*Na*_ (*g*_*Na*_), *I*_*Kr*_ (*g*_*Kr*_) and calcium channels (*I*_*CaL*_, *I*_*CaNa*_ and *I*_*CaK*_) through the PCa variable as previously reported in [7].
- A 6-well MEA device (Multichannel Systems) with 9 electrodes per well (60-6wellMEA20030iR-Ti [37]) where the corresponding finite element mesh is presented in the left panel of Fig 3. A cell heterogeneity field was applied on this finite element mesh following the strategy developed in section Heterogeneity.
- The sparse optimization was performed on a dataset of 1520 data points (76 first compounds, each compound simulated 20 times with different heterogeneities and sources). The FP traces corresponding to the last 10 compounds were also simulated 20 times with different heterogeneities and source, but used for the validation set. The same process was done for the calcium transient signals. The dictionary entries used for this classification problem are summarized in Table D in S1 Text.

### Results of TdP classification

**Fig 12:**
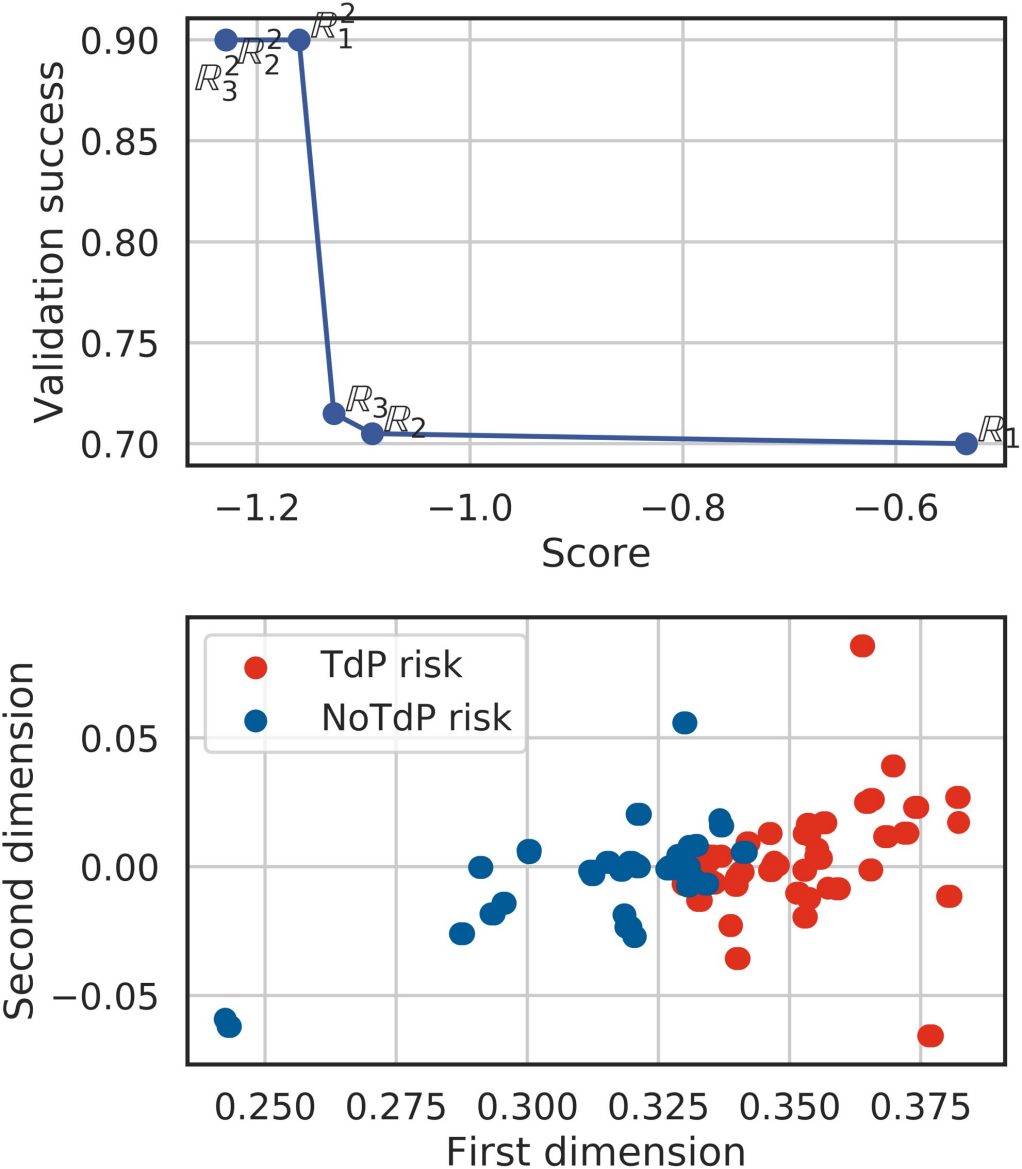
Section TdP classification: TdP risk classification through simulations of 86 compounds. Left: Validation versus Cost curve depending on the number of components and the dimension. Right: Drug repartition in the input space after convergence of the algorithm.

We start this section by commenting on the results of the classifier as function of the input space constructed by the greedy algorithm. In Fig 12 the success rate of the classification for the validation set is plotted as function of the cost presented in section Classification optimization, Eq (11). The cost minimised by the proposed algorithm is a pertinent descriptor of the success rate of the classifier. The input space selected by progressively increasing the input space dimension as well as the components per dimension produces a high success rate. The input space corresponding to the case where the input is in $R^{2}$ (with three dictionary components per direction) is shown in Fig 12, from which we can appreciate that the separation between the classes is satisfactory.

**Fig 13:**
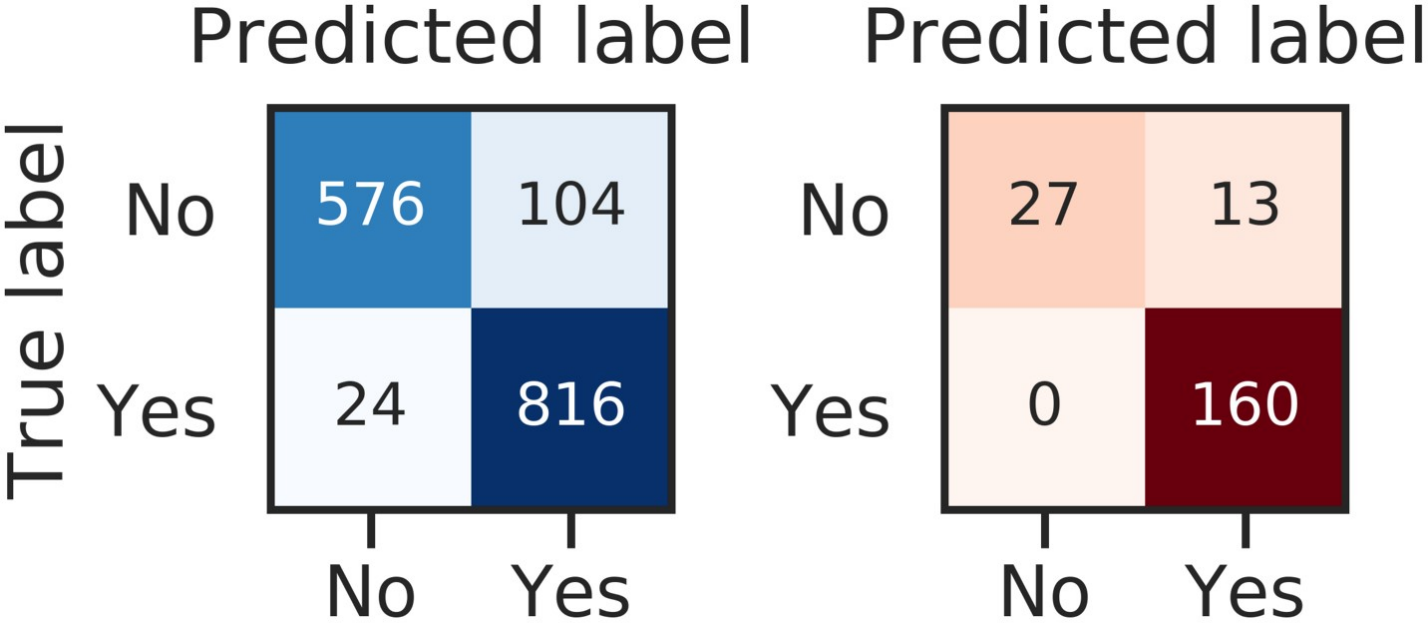
Section TdP classification: Confusion matrices obtained for TdP risk classification of 86 compounds after convergence of the algorithm. **Yes: TdP risk**. No: No TdP risk. Left: Training set (sample size: 1520) using randomized K-fold cross-validation. Sensitivity = 0.98, Specificity = 0.85 and Accuracy = 0.92. Right: Validation set (sample size: 200). Sensitivity = 1, Specificity = 0.675 and Accuracy = 0.935.

The results of the classification are detailed. In Fig 13 the confusion matrices for the training set (left, in blue) and for the validation set (right, red) are shown. Globally, the results are similar for training and validation (no apparent overfitting phenomena were seen). The type II error (wrongly classifying a compound as non-torsadogenic) is well minimized thanks to the choice to penalize false positives (*α* = 2 in the cost function, Eq 11). In the validation set, no compounds were wrongly classified as non-torsadogenic. Only the Propranolol was misclassified as torsadogenic (see Table E in S1 Text).

It is interesting to monitor the classification results at different stages of the algorithm. The confusion matrices are given in Figure A in S1 Text. Confusion matrices obtained for test and validation sets show an improved TdP risk classification when we increase the number of components and dimensions. This improvement is particularly visible on the test set for the first components. The training on the first dimension is not sufficient to classify well the validation set. Meaning that other dictionary entries would have been selected by the algorithm (for the first dimension) if the validation set was in the training set. However, dictionary entries selected for the second dimension seem to be better to discriminate torsadogenic risk on the validation set.

### Channel classification

This section is dedicated to the channel classification of 12 compounds based on *in vitro* data derived from MEA recordings of spontaneous beating hiPSC-CMs (Pluricyte Cardiomyocytes) cultured on 96 well MEA plates (8 electrodes per well, Axion Biosystems), as described in section Cell culture. As we are limited by the experimental sample size (see compound list in Table 1), we enriched the experimental database with a simulated database (for which we know the classification output). For this study only FP traces were recorded and used for the training and classification, no calcium transient measurements were performed.

#### Tests setup

**Fig 14:**
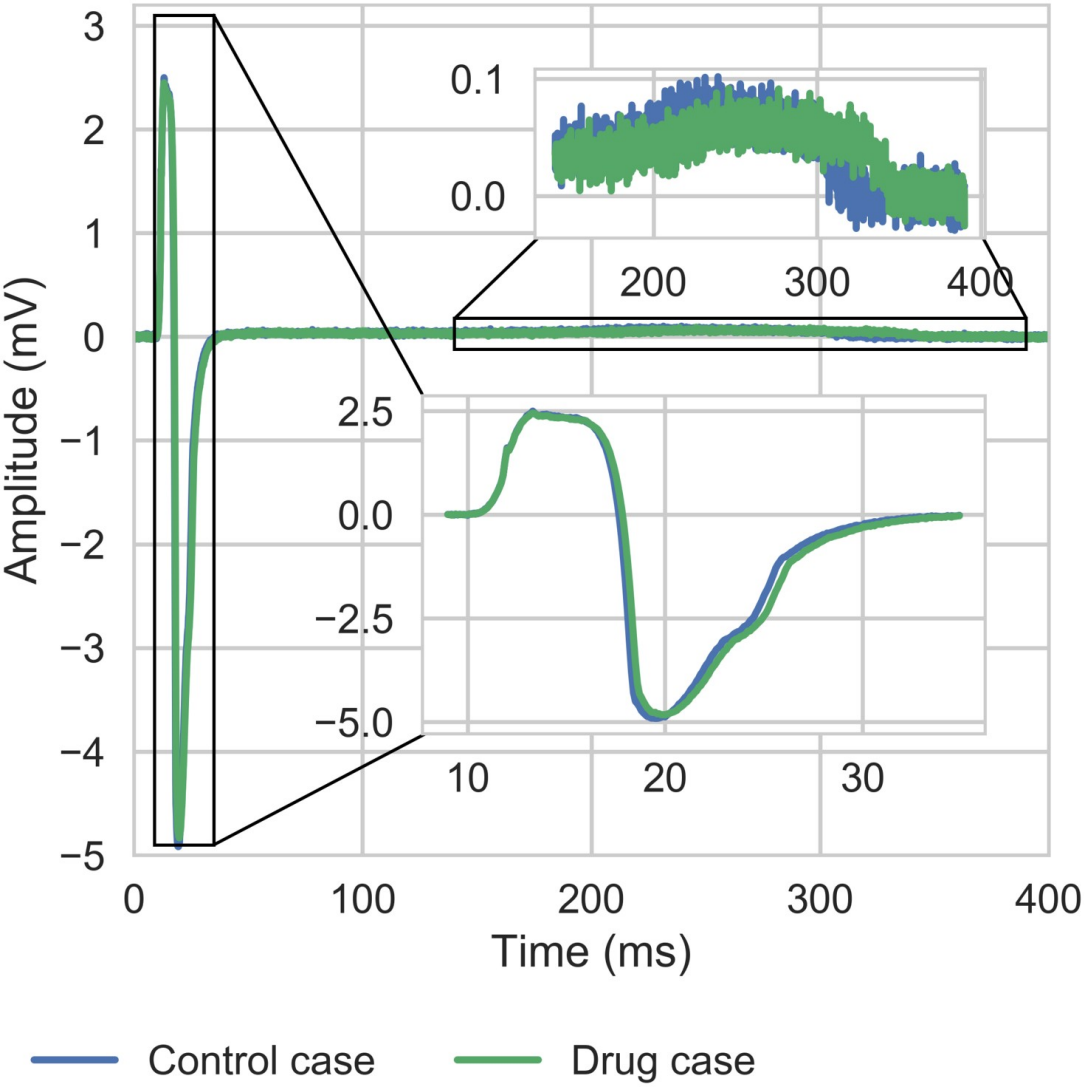
Section Channel classification: Simulated FP under control and compound conditions. FP trace from one electrode, showing the effect of drug simulation blocking the sodium channel at 4%, calcium channels at 3.6% and potassium channel at 27.9%.

The numerical choices leading to the results are summarised hereafter:

- Bidomain equation parameters are summarized in Table C in S1 Text.
- Drugs were modeled using Eq 11 presented in section MEA computational model. The *in silico* database was generated blocking alternatively sodium (*g*_*Na*_), potassium (*g*_*Kr*_) or calcium (PCa) channels of the ORd model at a random percentage between 0% and 50%. Other channels are blocked between 0% to 5% to introduce some variability (e.g. blocking sodium at 35%, calcium at 2% and potassium at 3.5%). An example is shown in Fig 14.
- The simulated sample size is 140 (computed from signals resulting from the simulation performed for different heterogeneity fields).
- A 96-well MEA device (Axion Bioystems) with 8 electrodes per well where the corresponding finite element mesh is presented in the right panel of Fig 3. A cell heterogeneity field was applied on this finite element mesh following the strategy developed in section Heterogeneity.

The experimental data leading to the results are summarised hereafter:

- *In vitro* data used for this part are FP traces recorded from a hiPSC-CM monolayer (Pluricyte Cardiomyocytes, Ncardia) plated on a 96 well MEA plate (8 electrodes per well) Axion Biosystems (Classic MEA 96 M768-KAP-96 [38]).
- The 12 “CiPA” compounds listed in Table 1 were tested on Pluricyte Cardiomyocytes and FP traces were recorded before and 30 minutes post compound addition. MEA results of 5 compounds were used for the training and MEA results of 7 “blind” compounds for the validation.
- Each compound was tested at 4 concentrations, 1 concentration per well and in 5 replicates (n = 5 per concentration).

The final experimental sample size was 75 for the training set and 85 for the validation set (some wells were removed from the analysis due to quiescence or noisy signal observations). The dictionary entry list is given in Table D in S1 Text.

Using the conductance-block model described in Eq 7 we obtain the percentage of activity for each channel and concentration. This is shown in Table G in S1 Text.

Two different kinds of classification problems have been studied. A binary classification (*i.e*. given a channel, is the molecule affecting its functioning), whose results are shown in section Binary classification: and a ternary classification (*i.e*. is the molecule affecting potassium, calcium or sodium?), whose results are reported in section Ternary classification. For the numerical experiments proposed, the success rate of the classifier for the training set was about 90%. In the following, we present in details the results on the *in vitro* data in the validation set.

#### Binary classification

**Fig 15:**
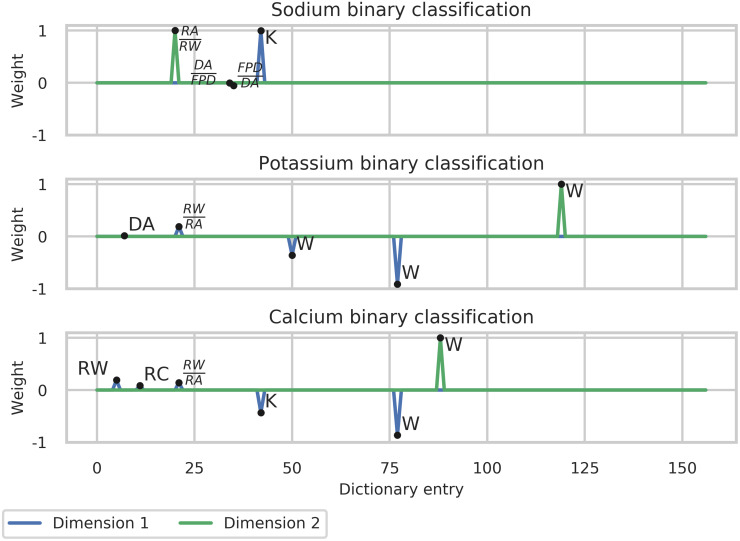
Section Channel classification (Binary classification part): Weights obtained by the optimised classification algorithm.

We start this section by describing the outcome of the greedy algorithm selection. These are shown, for the three classification problems addressed, in Fig 15, in which the weights of the dictionary entries are plotted. The selected entries are different (also in number) for the different classification problems. For instance, for the sodium binary classification, we obtained 3 components for the dimension 1 whereas for the same dimension, we obtained 4 components for the potassium case and 5 for the calcium case. In all the cases, the linear combinations retained are sparse.

The classification results are reported hereafter. First, an aggregated result is presented (considering all the different concentrations, providing an overall label). Then, in the last part of this section, the results at different concentrations are described.

#### Binary classification: Aggregated result

**Fig 16:**
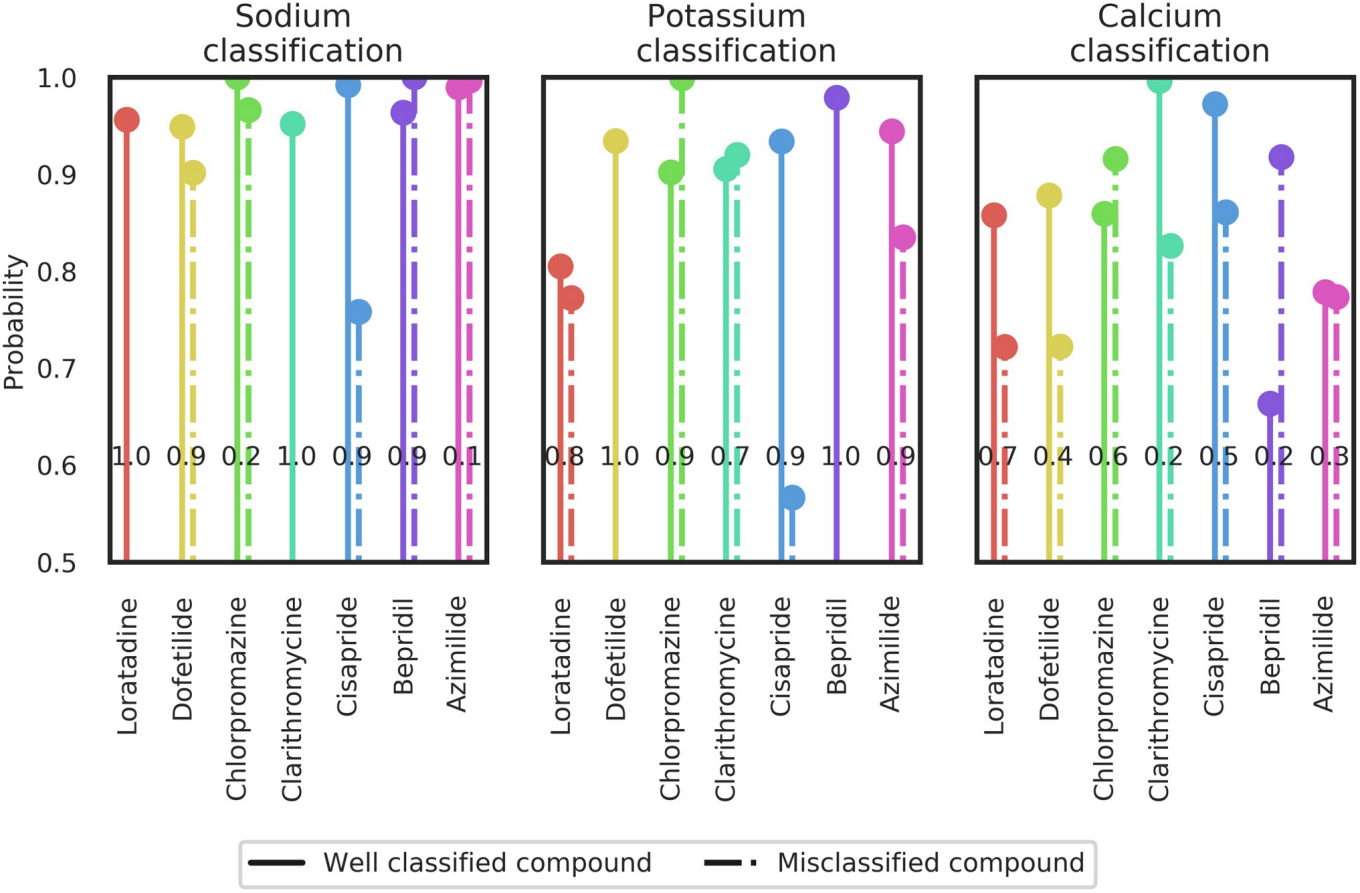
Section Channel classification (Binary classification part): Experimental data classification in binary case. Plain (resp. dotted) lines correspond to the average confidence of the LDA classifier for well classified (resp. misclassified) compound (well classification is according to Table 1). The black values on the lines correspond to the proportion of well classified observations for each compound.

Fig 16 shows classification results for the seven compounds that were included in the validation set. The value shown for each compound corresponds to the success rate of classifying the compound correctly as a blocker or non-blocker for either the sodium, potassium or calcium channel according to their label (see Table 1).

The results for the 7 molecules in the validation set are commented.

**Fig 17:**
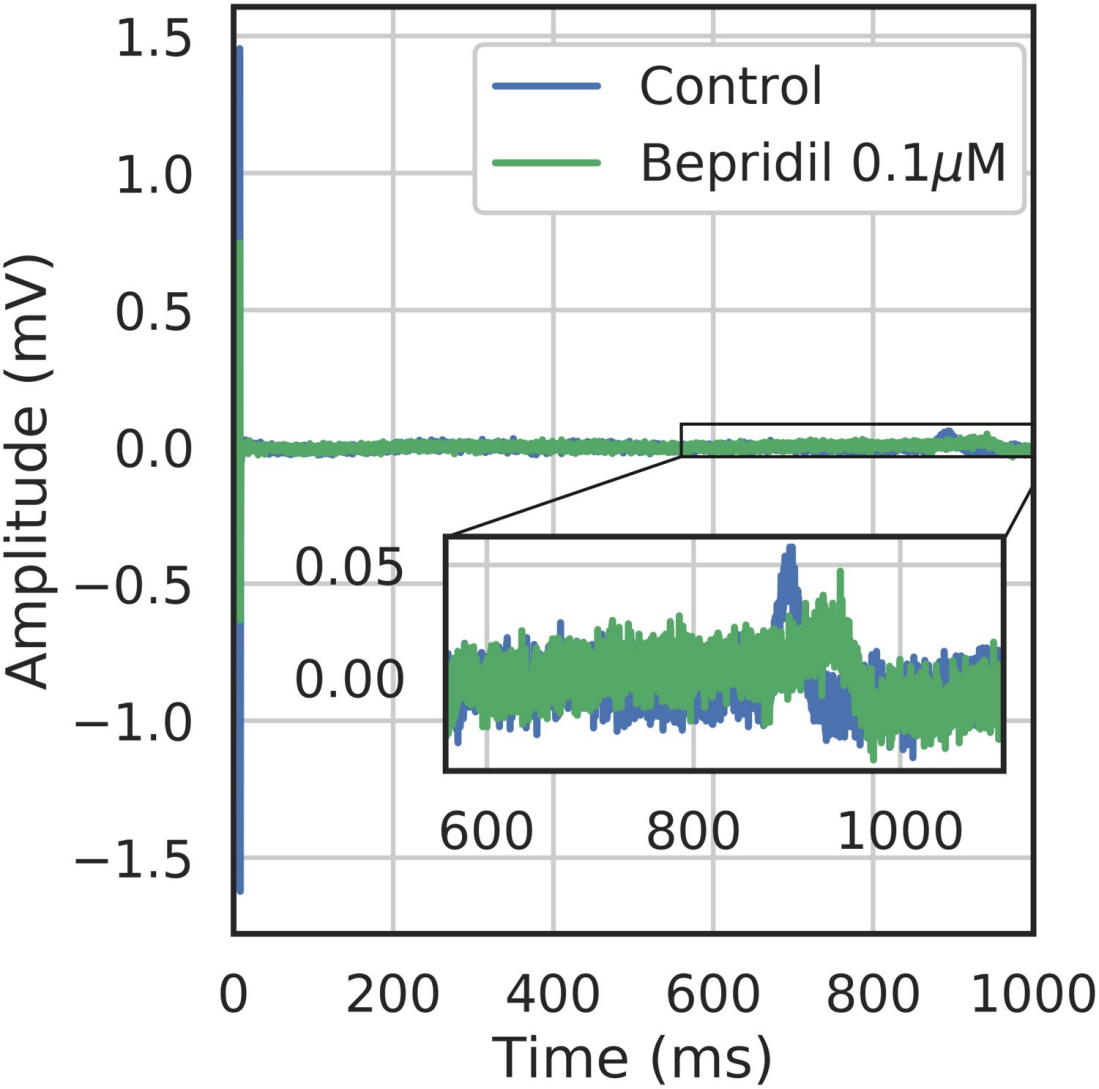
Section Channel classification (Binary classification part, Bepridil classification results): Example of experimental data with Bepridil, showing an increase in FPD and a decrease in DA of Pluricyte Cardiomyocytes.

1. ***Loratadine***: *potassium and calcium channel blocker* (Table 1)
  For the sodium channel block classification, Loratadine is always well classified (as a non sodium channel blocker) with high confidence (averaged probability returned by the classifier is close to one, see Fig 16). For potassium blockade, Loratadine is well classified in 80% of the cases. Moreover, when Loratadine is well classified, the classifier is more confident (> 0.8) than when it is misclassified (< 0.8).
2. ***Dofetilide***: *potassium channel blocker* (Table 1)
  The classifier always returns Dofetilide as a potassium channel blocker with a high probability. Dofetilide is also classified as a sodium blocker, but only for 10% of the cases and with a lower probability than when it is not classified as a sodium blocker. For the calcium channel block classification, Dofetilide is considered as a non-calcium channel blocker for 40% of the cases but with a higher probability than when it is considered as a calcium channel blocker.
3. ***Chlorpromazine***: *potassium, calcium and sodium channel blocker* (Table 1)
  Chlorpromazine is well classified for the potassium and calcium channel classifications (i.e. it is considered as a potassium channel blocker and calcium channel blocker). The success rates for Chlorpromazine are similar to those obtained with Loratadine. An explanation might be the fact that they approximately have the same factor between the hERG and Cav1.2 IC50 values. In addition, Chlorpromazine is classified as a sodium channel blocker in only 20% of all cases, but with a probability of 100%.
4. ***Clarithromycine***: *potassium channel blocker* (Table 1)
  Clarithromycine is well considered as a non-sodium channel blocker with a high confidence and for all tested samples. In 70% of the cases Clarithromycine is well classified as a potassium channel blocker with around 90% of confidence. However, Clarithromycine is also labeled as a calcium blocker for 80% of the samples and with more than 80% of confidence. Important to note here is that, although Clarithromycine is labeled as a non-calcium channel blocker for only 20% of the samples, the confidence for this well classification is close to 100%.
5. ***Cisapride***: *potassium channel blocker* (Table 1)
  If we compare classification results obtained for Chlorpromazine and Cisapride, the potassium channel binary classification success rate is the same. However, the classifier is more confident when Cisapride is well classified. Moreover, for the calcium channel classification, Chlorpromazine is classified as a calcium channel blocker in 60% of the cases whereas Cisapride is classified as non-calcium channel blocker in 50% of the cases with a higher confidence than when Cisapride is misclassified as calcium channel blocker. Moreover, in 90% of the samples tested, Cisapride is being classified as a non-sodium channel blocker with a confidence close to 100%. These results are in good agreement with the high potency of Cisapride to block the hERG channel, and the multi-channel block ability (hERG, Nav1.5 and Cav1.2) for Chlorpromazine.
6. ***Bepridil***: *potassium, calcium and sodium channel blocker* (Table 1)
  Sodium and potassium channel blockade classification for Bepridil is well captured by the classifier (with high proportion and high confidence). Calcium channel blockade is not seen by the classifier for Bepridil. A potential explanation could be that if calcium and potassium channels are blocked simultaneously, Bepridil does not show a specific pattern of a calcium channel blocker, but essentially potassium and sodium channel patterns are detected as shown in Fig 17 (e.g FPD prolongation due to potassium channel block and DA decrease due to sodium channel block).
7. ***Azimilide***: *potassium, sodium and calcium channel blocker* (Table 1)
  Azimilide is well classified as a potassium channel blocker with a high confidence and for 90% of the sample. The sodium channel blockade by Azimilide is clearly not seen by the classifier as 90% of the sample is labeled as non-sodium channel blockade with a confidence close to 100%. The calcium channel blockade classification is also less clear as only 70% of the samples are labeled as non-calcium channel blockade with almost 80% of confidence. This could be related to the potency of Azimilide to block the inward sodium currents and L-type calcium channels is lower than for blocking the hERG channel [52]. Besides, the highest concentration tested was lower than the IC50 values for blocking sodium and calcium channels (Table 1). A dictionary entry chosen by the algorithm for potassium and calcium blockade classification is the ratio $\frac{RW}{RA}$ (see Fig 15). As shown in [53], hERG channel block can induce a T-wave flattening in the ECG. This phenomenon is also observed in the FP repolarization of Pluricyte Cardiomyocytes for 0.1*μM* of Bepridil (see Fig 17) and could be an explanation of the $\frac{RW}{RA}$ selection by the algorithm.

For most of the cases, drugs are well classified with a high confidence. However, this is not always the case. For instance, Dofetilide has been perfectly classified as a potassium channel blocker with a high confidence (around 90%), but Dofetilide has also been misclassified as a calcium channel blocker with a high confidence (around 70%).

#### Binary classification: Study for each concentration

**Fig 18:**
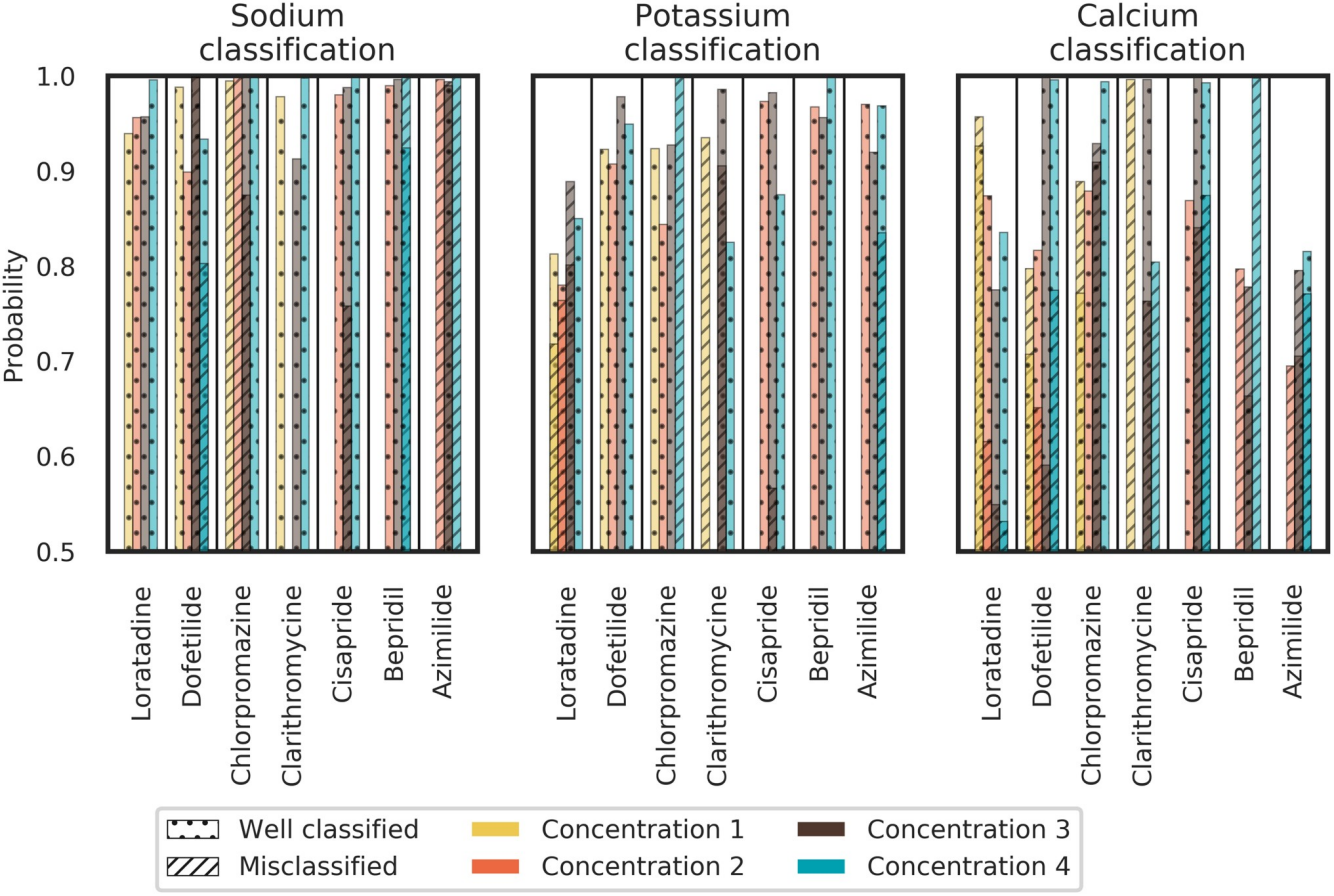
Section Channel classification, Binary classification part: Experimental data classification in binary case for each concentration. Some concentrations were not used due to the quiescence or noisy signal observation. For each concentration, the LDA classifier returns the average probability for well classified (dotted bars) and misclassified (hatched bars) compounds.

Details for ion channel block classification of each concentration of each compound are given in Fig 18. This figure shows how each compound was classified at each concentration. The interest is to study the evolution of the classification with respect to increasing concentrations.

As done in the previous section, we present the results for each of the 7 molecules in the validation set.

1. ***Loratadine***: *potassium and calcium channel blocker* (Table 1)
  We know from Fig 16 that Loratadine is always classified as a non-sodium channel blocker. From Fig 18 we can conclude that the confidence of Loratadine being a non-sodium channel blocker increases with higher concentrations. In addition, Loratadine has also been classified as a potassium channel blocker in 80% of the cases (see Fig 16). Fig 18 shows that the classification is the best at the highest concentration (no misclassification), which can be explained by the relatively low test concentrations compared to the IC50 values (Table 1). Moreover, for the first two concentrations in the potassium channel classification, the confidence is higher when Loratadine is well classified than when Loratadine is misclassified. A bad mark is the increase of the misclassified confidence for the first three concentrations. However, for the highest concentration tested, none of the samples were misclassified. Concerning the classification for the calcium channel, the success rate of Loratadine to be classified as a calcium channel blocker was 70% (Fig 16); and based on Fig 18 we can conclude that the misclassified confidence decreases strongly when the concentration increases. This is in line with the differences seen in IC50 values between hERG, Cav1.2 and Nav1.5 (Table 1).
2. ***Dofetilide***: *potassium channel blocker* (Table 1)
  Dofetilide was wrongly labeled as a calcium channel blocker in 60% of the cases (see Fig 16). However, the well-classified confidence increases strongly with the concentration (see Fig 18), which means that the confidence of Dofetilide being a calcium channel blocker decreases when the concentration increases. The well-classified probability for the sodium channel (Dofetilide being a non-sodium channel blocker) and potassium channel (Dofetilide being a potassium channel blocker) is around 90% or even higher for all concentrations tested.
3. ***Chlorpromazine***: *potassium, calcium and sodium channel blocker* (Table 1)
  Chlorpromazine is known to block sodium, potassium and calcium channels (see Table 1). Only for the first three concentrations, Chlorpromazine is clearly seen as a potassium channel blocker (Fig 18). The fourth and highest concentration show that sodium and calcium channels are affected instead of potassium. This is in line with the different potencies of Chlorpromazine for the different ion channels: Chlorpromazine blocks hERG more potently than sodium or calcium (see Table 1). The calcium channel blockade is confirmed by the fact that well-classified confidence for calcium channel block increases with concentration, in addition to being well classified in 60% of all cases.
4. ***Clarithromycine***: *potassium channel blocker* (Table 1)
  Clarithromycine is better classified as a potassium channel blocker at higher concentrations (higher confidence for the third concentration and no misclassification for the fourth concentration). Also the sodium classifier shows us that for all test concentrations, Clarithromycine is well classified as a non-sodium channel blocker. However, for any concentration, the calcium classifier does not give us satisfactory results, which means that Clarithromycine is wrongly classified as a calcium channel blocker.
5. ***Cisapride***: *potassium channel blocker* (Table 1)
  Well-classified confidence for Cisapride is always higher than misclassified confidence regardless of the concentration and, particularly for the sodium and potassium channel classifiers. This is in line with Cisapride being a very potent potassium blocker (see Table 1).
6. ***Bepridil***: *potassium, calcium and sodium channel blocker* (Table 1)
  Bepridil is well classified as a sodium and potassium channel blocker with a high confidence. This is not the case for the calcium classification. An explanation could be that the potassium channel blockade hides the effect of the calcium channel blockade as above mentioned.
7. ***Azimilide***: *potassium, sodium and calcium channel blocker* (Table 1)
  Azimilide is classified as a potassium channel blocker with a probability higher than 90% for all concentrations tested. However Azimilide is misclassified for sodium and calcium channel blockade. As abovementioned, this could be related to the fact that Azimilide blocks the inward sodium currents and L-type calcium channels at concentrations 5-10 times higher than required for blocking the hERG channel [52].

#### Ternary classification

**Fig 19:**
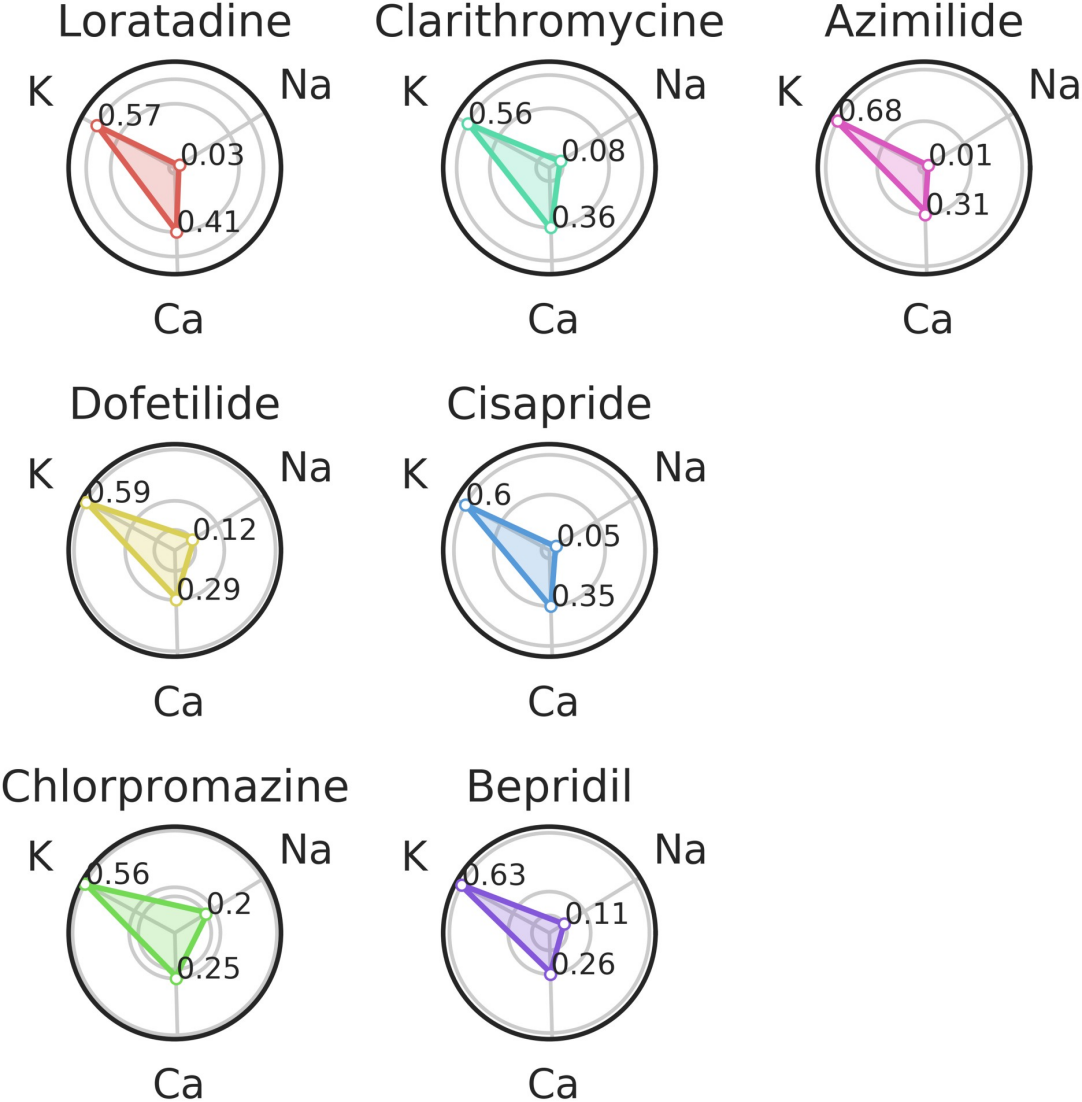
Section Ternary classification (Ternary classification part): Experimental data classification in ternary case.

For the ternary classification we only considered one classifier but with three outputs: sodium, potassium and calcium channel blockers. Aggregated results for the ternary classification are presented in Fig 19.

The ternary classifier classified all seven compounds from the validation set as potassium channel blockers.

**Fig 20:**
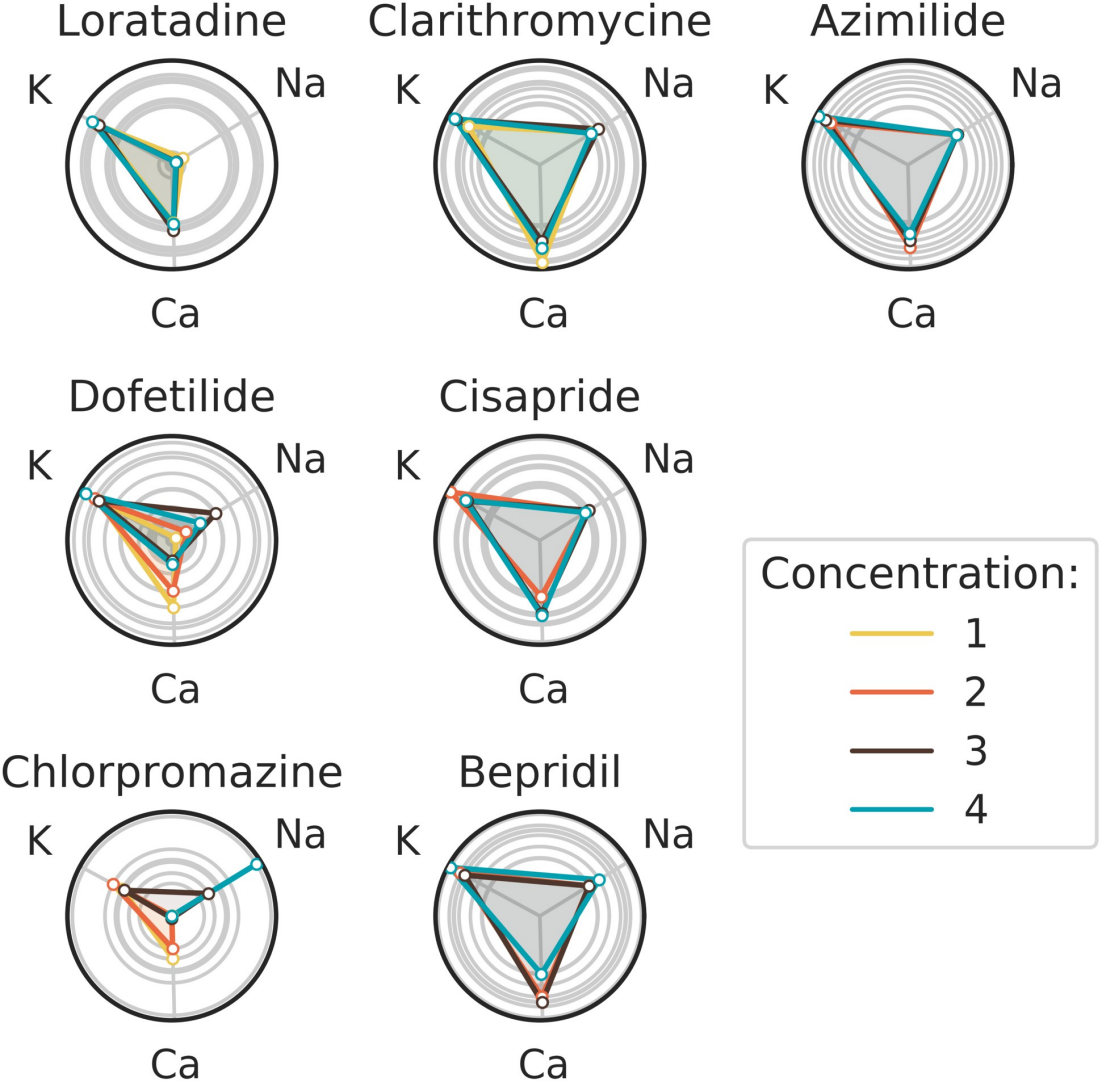
Section Ternary classification (Ternary classification part): Experimental data classification in ternary case for each concentration.

As expected, the probability returned by the classifier decreases when the IC50 value increases (for example the probability for Loratadine to be a calcium channel blocker is 0.41 with *IC*50 = 11.4*μM* (see Table 1) and the probability for Dofetilide to be a calcium channel blocker is 0.29 with *IC*50 = 26.7*μM* (see Table 1)). These results do not take into account the different concentrations tested. The probabilities given for each concentration are given in Fig 20.

The results for each of the 7 molecules are detailed hereafter. For sake of brevity, both aggregated and by-concentration results are commented.

**Fig 21:**
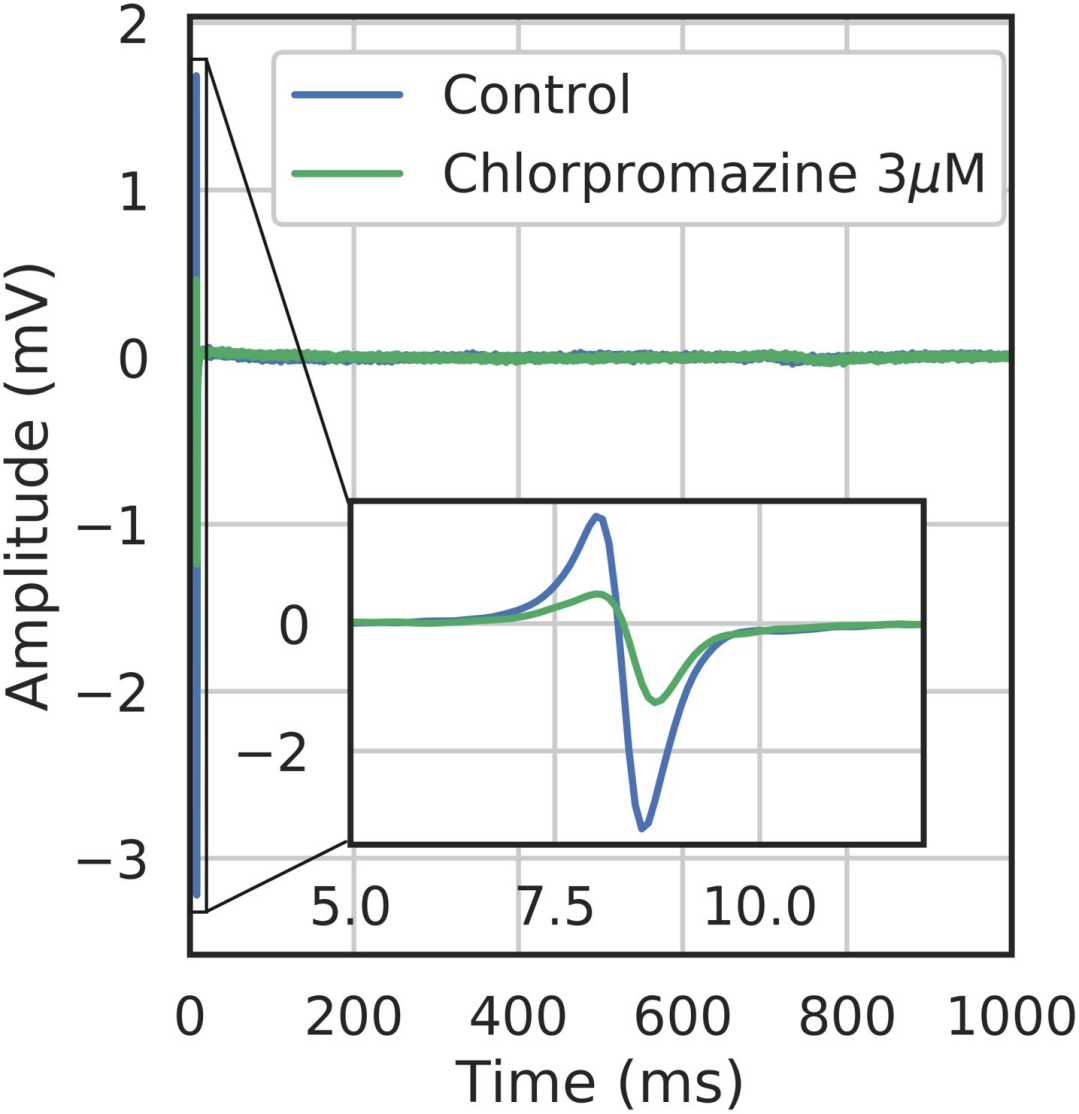
Section Channel classification (Ternary classification part, Chlorpromazine classification results): Example of experimental FP trace with Chlorpromazine.

1. ***Loratadine***: *potassium and calcium channel blocker* (Table 1)
  Loratadine is well classified as a potassium channel blocker with a probability equals to 0.57 (Fig 19). The second highest probabilty concerns the calcium channel blocker, which was expected as Loratadine is more potent to block hERG (6.1*μM*, see Table 1) than to block the L-type calcium channel (11.4*μM*, see Table 1). Fig 20 shows us that the confidence of the classifier is almost the same regardless to the concentration.
2. ***Dofetilide***: *potassium channel blocker* (Table 1)
  From Fig 19, we can see that Dofetilide is well classified as a potassium channel blocker with a probability equals to 0.59. If we now look at the classification results of Dofetilide in Fig 20, a high concentration gives a higher probability of being a potassium channel blocker and a lower probability to be a calcium channel blocker (which is in line with the binary classification method presented in Fig 17). This can be explained by the fact that for the three lower concentrations, the potassium activity is always higher than 90%(see Table G in S1 Text) whereas the highest concentration corresponds to a 75% activity (see Table G in S1 Text).
3. ***Chlorpromazine***: *potassium, calcium and sodium channel blocker* (Table 1)
  Chlorpromazine is well classified as a potassium channel blocker with a probability equals to 0.56 (Fig 19). The probabilities for Chlorpromazine of being a calcium or sodium channel blocker are close to each other (Fig 19), which was expected as the IC50 values for calcium and sodium channel blockade are close to each other as well (Table 1). The confidence to classify Chlorpromazine as a sodium channel blocker is the highest for the highest concentration tested (3*μM*, see Table 1) (Fig 20). An explanation of this result could be that some compensation effects would appear on the repolarization due to the simultaneous block of potassium as well as calcium. 3*μM* of Chlorpromazine corresponds at a 50% activity of the sodium channel (see conductance-block model in section Drug modeling), which is clearly visible on the depolarization amplitude (see Fig 21).
4. ***Clarithromycine***: *potassium channel blocker* (Table 1)
  Clarithromycine is well classified as a potassium channel blocker with a probability equals to 0.56. It is interesting to see that the confidence of being a calcium channel blocker is lower for Clarithromycine than for Loratadine (see Fig 19). This point is expected because Loratadine is known to block calcium channels with an IC50 of 11.4*μM* (see Table 1), which is not the case for Clarithromycine (IC50 > 30*μM*). Another good point is that the confidence of being a potassium channel blocker for Clarithromycine slightly increases with higher concentrations (Fig 20).
5. ***Cisapride***: *potassium channel blocker* (Table 1)
  Cisapride is well classified as a potassium channel blocker with a probability equals to 0.6 (see Fig 19). The second highest confidence is for the calcium channel blocker. These channel blockade probabilities are in good agreement with the IC50 values of Cisapride for potassium channel blockade (0.02*μM*) and calcium channel blockade (11.8*μM*) (Table 1). The difference in these values might also explain the observation that the confidence of being a potassium channel blocker decreases when the concentration increases, following by a higher confidence of Cisapride being a calcium channel blocker (Fig 20).
6. ***Bepridil***: *potassium, calcium and sodium channel blocker* (Table 1)
  Bepridil is well classified as a potassium channel blocker with a probability equals to 0.63 (see Fig 19). The order of the different ion channel blockade probabilities is in good agreement with the IC50 values order (Table 1). The sodium channel blockade probability is 0.11. This probability is coherent in the sense that Bepidril is known to block the sodium channel; other compounds which are not known as sodium channel blockers have a lower probability (0.01-0.08) of being a sodium channel blocker (except for Dofetilide at low concentrations).
  Unexpectedly, Fig 19 shows that the probability to be a calcium channel blocker is similar between Bepridil and Dofetilide (not a calcium channel blocker). Even for the last concentration of Bepridil, there is a decreasing confidence of being a calcium channel blocker in favor of being a potassium and sodium channel blocker (Fig 20). This could be explained by the fact that Bepridil has a higher potency for blocking hERG compared to blocking calcium channels and that the effects of hERG channel blockade masked the effects of blocking calcium channels.
7. ***Azimilide***: *potassium, sodium and calcium channel blocker* (Table 1)
  Azimilide is well classified as a potassium blocker with a probability of 0.68 (see Fig 19). Although it is known that the potency of Azimilide to block the inward sodium currents and L-type calcium channels is lower than blocking the hERG channel, the probability of being a sodium channel blocker was still lower than expected and did not change with higher concentrations.

## Discussion

Human iPSC-CMs are being increasingly adapted as a novel in vitro model to better recapitulate human heart function and to complement or replace existing in vitro assays for improved cardiac safety assessment. Of the many studies that have now investigated the impact of drugs on the electrophysiology of hiPSC-CMs, the most well-known is the multisite CiPA initiative. Data presented in [6] describe the utility of hiPSC-CMs in combination with MEA and voltage-sensing optical methods in evaluating the electrophysiological responses to 28 drugs linked to low, intermediate, and high TdP risk categories. Studies like the CiPA multisite study show promising results. However, predicting TdP risk at a reasonable level of accuracy remains a challenge. Besides, many screening platforms, like various MEA and calcium-flux devices, are becoming increasingly sophisticated and generate large multidimensional datasets. Improved automated analysis methods, including classification methods to accurately predict the risk for ion-channel block and TdP, are needed.

In the present work, a preliminary step towards the setup of high-throughput screening procedures was attempted. In particular, a method was proposed to systematically deal with classification problems involving “CiPA” compounds for their risk to induce TdP as well as for their ion channel blocking properties.

### Algorithm

This algorithm selects and combines pertinent features extracted from the signals in order to maximize the classification score (both in terms of the success rate and the confidence of the classifier) by means of a double greedy optimization. The algorithm promotes sparsity (hence mitigating the overfitting risk) and it is fully scalable in terms of parallelism (remark that the number of cores can potentially equal the dictionary entry size). In this paper, the input space computed by the algorithm maximize a score by linearly separating the classes samples, using the classical LDA method. It would be interesting to test the algorithm with other classifiers such as support vector machine (SVM) with different kernels or k nearest neighbor (KNN) and against classification with PCA.

We applied the algorithm on simulated FP and calcium transient data for TdP risk classification as well as on *in vitro* data coming from FP signals recorded from hiPSC-CMs (Pluricyte Cardiomyocytes) that were cultured on 96 well MEA plates and subjected to 12 CiPA reference compounds (5 compounds were used as a training set and 7 were used to validate the algorithm).

### TdP risk assessment

The classifiers obtained have given encouraging results for a drug safety profile of the compounds. Compounds known to have a high TdP risk were 100% well classified according to the arrhythmogenicity risk classification and a compound known to have a low TdP risk was well classified in 67% of the cases. This is conforming to the fact that we decided to put a strong weight on the type II error (wrongly classify a compound as non-torsadogenic). Concerning the torsadogenicity classification, more tests have to be done with higher concentrations (10xEFTPC, 50xEFTPC, etc). Thus, the compound impact on physiological traces (FP and intracellular calcium transient) would be more important, which would improve the classification (bigger margin between the data points and the separation plan). However, even at EFTPC, the TdP risk classification results are encouraging as only Propranolol was misclassified as torsadogenic. Particularly, the algorithm allows us to weight the type II error. To improve the arrhythmogenicity assessment, a ternary classifier could be established to distinguish low, moderate or high TdP risk.

### Ion-channel blockade

Concerning ion-channel blockade classification of compounds, potassium was always well classified with a high confidence. Moreover, for the ternary classification study, for most of the tested compounds, the lower the IC50 for a channel, the higher the confidence of the classifier to block this channel.

The binary sodium channel blockade classification is good for all the compounds except for Chlorpromazine (at low concentration). The ternary classification study shows similar probabilities of Chlorpromazine for blocking the sodium channel as for blocking the calcium channel, which is in agreement with the similar IC50 values of Chlorpromazine for these channels.

However, the binary classification is less good for the calcium channel blockade classification. This could also be related to the fact that all CiPA compounds from the validation set block the hERG channel and with higher potencies compared to blocking the calcium channel. The effect of blocking hERG could mask the effect of blocking calcium, making calcium channel blockade more difficult to classify.

In general, the binary and ternary classification strategies are in a good agreement (e.g potassium channel blockade is always well classified).

Nevertheless, more tests have to be done on the algorithm in order to validate and/or improve the classifiers.

For the channel block classification, simulations have been done only on highly pure channel block properties (no multi-channel blockade), simplified to only three types of channels: potassium, calcium or sodium, which is often not representative of the total ion channel blocking effects a compound could have. Training based on *in silico* multi-channel blockade would be more realistic and would most likely increase the robustness of the classification. Moreover, the present experimental protocol was performed at different concentrations for each compound. The dictionary entry could take this information into account.

Each application presented in this paper was based on one specific model of MEA device. It would also be interesting to know whether the MEA device might have an impact on the analysis of the drug effect, *i.e*. to study the case where we learn with one MEA device and we validate with data coming from another MEA device.

The addition of intracellular calcium transient data would increase the classification in order to identify not only effects on ion-channels but also to detect negative and positive inotropic effects thereby having the capability to classify other classes of compounds, such as calcium-sensitizers or adrenergic receptor agonists.

The time compound dynamic was not studied in this paper. The dictionary could be extended with new biomarkers as beat rate or depolarization standard deviation. These new entries could provide information on the impact of the compound on the monolayer stability. In order to represent this behavior in the *in silico* dataset, a pacemaker action potential model showing experimental beat rate behavior (Paci *et al* [54]) could be introduced.

In summary, the algorithm that we developed proved to be a promising tool to classify compounds for their risk to induce TdP as well as for their ion-channel blocking properties based on *in vitro* and in silico data derived from hiPSC-CMs. Therefore, this method can be implemented in *in vitro* MEA and/or calcium-flux studies using hiPSC-CMs where it may serve as a tool to improve machine learning approaches and to deliver fast and reliable prediction of drug-induced ion channel blockade and proarrhythmic behavior to advance cardiac safety assessment.

## Supporting information

S1 Text Demonstrations, parameters used, experimental data information and stopping criterion justification. (PDF)
