# Supplementary material for: A greedy classifier optimization strategy to assess ion channel blocking activity and pro-arrhythmia in hiPSC-cardiomyocytes
Source: PLoS Comput Biol. 2020 Sep 25;16(9):e1008203. doi: 10.1371/journal.pcbi.1008203 (PMC7549820; doi:10.1371/journal.pcbi.1008203)
Supplement: S1 Text — (PDF) [file pcbi.1008203.s001.pdf]

Supporting information

Cost function demonstration: 2 classes

In the perfect case, we have $l_i = \widehat{l_i}$ and $\widehat{p_i} = 1, \forall i$. Then,

$$s_{N_s} = -\frac{1}{N_s}\sum_{i=1}^{N_s}\Big[\frac{N_s}{n_1}\delta_1(\widehat{l_i})\delta_1(l_i) + \frac{N_s}{n_{-1}}\delta_{-1}(\widehat{l_i})\delta_{-1}(l_i)\Big].$$

$$\iff s_{N_s} = -\frac{1}{N_s}\sum_{i=1}^{N_s}\frac{N_s}{n_1}\delta_1(\widehat{l_i})\delta_1(l_i) - \frac{1}{N_s}\sum_{i=1}^{N_s}\frac{N_s}{n_{-1}}\delta_{-1}(\widehat{l_i})\delta_{-1}(l_i).$$

Moreover, we know that $l_i = 1$, n_1 times and $l_i = -1$, n_{-1} times. Finally, we find that the minimum value is $s_{N_s} = -2$. In the worst case, we have $l_i \neq \widehat{l_i}$ and $\widehat{p_i} = 1, \forall i$. Then,

$$s_{N_s} = -\frac{1}{N_s}\sum_{i=1}^{N_s}\Big[-\alpha\frac{N_s}{n_1}\delta_{-1}(\widehat{l_i})\delta_1(l_i) - \frac{N_s}{n_{-1}}\delta_1(\widehat{l_i})\delta_{-1}(l_i)\Big].$$

Moreover, we know that $l_i = 1$, n_1 times and $l_i = -1$, n_{-1} times. Then,

$$s_{N_s} = \frac{1}{N_s}\Big(\alpha\frac{N_s}{n_1}n_1 + \frac{N_s}{n_{-1}}n_{-1}\Big) \implies s_{N_s} = 1 + \alpha.$$

Cost function demonstration: general case

We have the following cost function:

$$s_{N_s} = -\frac{1}{N_s}\sum_{i=1}^{N_s}\widehat{p_i}\Bigg\{\sum_{j=1}^{k}\Big[\frac{N_s}{n_j}\delta_j(\widehat{l_i})\delta_j(l_i) - \frac{N_s}{n_j}\alpha_j\sum_{\substack{m=1\\m\neq j}}^{k}\delta_m(\widehat{l_i})\delta_j(l_i)\Big]\Bigg\}.$$

In the best case, we have $l_i = \widehat{l_i}$ and $\widehat{p_i} = 1, \forall i$. Then,

$$s_{N_s} = -\frac{1}{N_s}\sum_{i=1}^{N_s}\sum_{j=1}^{k}\frac{N_s}{n_j}\delta_j(\widehat{l_i})\delta_j(l_i).$$

We know how many samples are labeled for each class (n_1 for class 1 and n_2 for class 2). Then,

$$s_{N_s} = -\frac{1}{N_s}\Big(\frac{N_s n_1}{n_1} + \frac{N_s n_2}{n_2} + ... + \frac{N_s n_k}{n_k}\Big) \implies s_{N_s} = -k.$$

On the other hand, in the worst case, we have $l_i \neq \widehat{l_i}$ and $\widehat{p_i} = 1, \forall i$. Then,

$$s_{N_s} = -\frac{1}{N_s}\sum_{i=1}^{N_s}\Bigg\{\sum_{j=1}^{k}-\frac{N_s}{n_j}\alpha_j\sum_{\substack{m=1\\m\neq j}}^{k}\delta_m(\widehat{l_i})\delta_j(l_i)\Bigg\}.$$

$$\iff s_{N_s} = \frac{1}{N_s}\sum_{i=1}^{N_s}\sum_{j=1}^{k}\frac{N_s}{n_j}\alpha_j\sum_{\substack{m=1\\m\neq j}}^{k}\delta_m(\widehat{l_i})\delta_j(l_i).$$

For the same reasons concerning the number of labels in each class, we have:

$$s_{N_s} = \frac{1}{N_s}\Big(\frac{N_s}{n_1}\alpha_1 n_1 + \frac{N_s}{n_2}\alpha_2 n_2 + ...\frac{N_s}{n_k}\alpha_k n_k\Big) \implies s_{N_s} = \sum_{j=1}^{k}\alpha_j.$$

Bidomain and electrode model parameters

C_{el}	R_i	R_{el}
$1nF$	$2M\Omega$	$10M\Omega$

Table A. Parameters used for the imperfect electrode model.

A_m	C_m	σ_i	σ_e
$200cm^{-1}$	$1.0\mu Fcm^{-2}$	$5.0nScm^{-2}$	$5.0nScm^{-2}$

Table B. Bidomain equation parameters used for Multichannel Systems MEA device.

A_m	C_m	σ_i	σ_e
$1200cm^{-1}$	$1.0\mu Fcm^{-2}$	$1.2\mu Scm^{-2}$	$1.2\mu Scm^{-2}$

Table C. Bidomain equation parameters used for Axion MEA device with Pluricyte Cardiomyocytes cell line.

Field Potential Biomarkers computation

In this section, the computation of the biomarkers from FP time series is detailed. Let y be a FP signal. We defined as depolarization part (t_1, y_1) from $t = 0$ to $t = 100ms$ and the repolarization part (t_2, y_2) from $t = 100$ to $t = 1200ms$.

DA (Depolarization Amplitude):

Difference between the maximum and minimum value of the FP during the depolarization.

$$DA = max(y_1) - min(y_1). \quad (1)$$

RA (Repolarization Amplitude):

Maximum in absolute value of the repolarization.

$$RA = max(|y_2|). \quad (2)$$

FPD (Field Potential Duration):

Time difference between RA and the maximum in absolute value of the depolarization.

For the depolarization:

$$t_{dep} = t\left[\underset{t}{\operatorname{argmax}}(|y_1(t)|)\right]. \quad (3)$$

For the repolarization:

$$t_{rep} = t\left[\underset{t}{\operatorname{argmax}}(|y_2(t)|)\right]. \quad (4)$$

Then,

$$FPD = t_{rep} - t_{dep}. \quad (5)$$

AUC_r (Area Under Curve of the repolarization wave)

To get the repolarization, y_2 is truncated around $\pm\Delta t$ of t_{rep}. We used $\Delta t = 100$ms. The trapezoidal rule is used to approximate the integral.

$$\text{AUCr} = \left|\int_{t_{rep}-\Delta t}^{t_{rep}+\Delta t} y_2(t)\text{d}t\right|. \quad (6)$$

RC (Repolarization Center)

Offset of the barycenter (with respect to time) of the repolarization wave.

$$\mathrm{RC} = \int_{t_{rep}-\Delta t}^{t_{rep}+\Delta t} t\bar{y}_2(t)\mathrm{d}t - t_{dep}. \tag{7}$$

With $\bar{y}_2(t)$ a rescaling such that it is strictly positive and integrates to 1 on $[t_{rep} - \Delta t, t_{rep} + \Delta t]$.

RW (Repolarization Width)

Standard deviation of the repolarization wave.

$$\mathrm{RW} = \left[\int_{t_{rep}-\Delta t}^{t_{rep}+\Delta t} t^2\bar{y}_2(t)\mathrm{d}t - \left(\int_{t_{rep}-\Delta t}^{t_{rep}+\Delta t} t\bar{y}_2(t)\mathrm{d}t\right)^2\right]^{1/2}. \tag{8}$$

FPN (FP Notch)

Potential value $4ms$ after t_{dep}. To be less sensitive to noise, the signal is multiplied by a test function $\phi(t_1) = \exp\left[-\frac{(t_1-(t_{dep}+4))^2}{0.04}\right]$.

$$\mathrm{FPN} = \int_{t_1} y_1(t_1)\phi(t_1)\mathrm{d}t_1. \tag{9}$$

Calcium Signals Biomarkers computation

In this section, the computation of the biomarkers from intracellular calcium concentration time series is detailed. Let y be the intracellular calcium concentration signal.

CA (Calcium Amplitude):

Difference between the maximum and minimum value of the signal.

$$CA = max(y) - min(y). \tag{10}$$

DC (Drowsing Calcium):

Corresponding to the resting calcium, computed as the minimum value of the signal.

$$DC = min(y). \tag{11}$$

CDX (Calcium Duration):

Similarly to APD, CDX is the time interval corresponding to $X\%$ repolarization. Let denote by y_1 the signal from $t = 0ms$ to $t = t\left[\underset{t}{\mathrm{argmax}}(|y(t)|)\right] ms$ and y_2 the signal from $t = t\left[\underset{t}{\mathrm{argmax}}(|y(t)|)\right] ms$ to $t = 1200ms$.

For the depolarization:

$$t_{dep} = t\left[\underset{t}{\mathrm{argmin}}(|y_1(t) - \frac{100-X}{100}CA + DC|)\right]. \tag{12}$$

For the repolarization:

$$t_{rep} = t\left[\underset{t}{\mathrm{argmin}}(|y_2(t) - \frac{100-X}{100}CA + DC|)\right]. \tag{13}$$

Then,

$$CDX = t_{rep} - t_{dep}. \tag{14}$$

Index (electrodes median)	Index (electrodes mean)	Index (electrodes max)	Index	Entry name
0	7			DA
1	8			RA
2	9			FPD
3	10			AUC_r
4	11			RC
5	12			RW
6	13			FPN
14	22	30		RA/DA
15	23	31		DA/RA
16	24	32		RA/FPD
17	25	33		FPD/RA
18	26	34		DA/FPD
19	27	35		FPD/DA
20	28	36		RA/RW
21	29	37		RW/RA
			38	CD90
			39	CD75
			40	CD50
			41	CD25
			42	CA
			43	DC
			44	AUC90
			45	AUC75
			46	AUC50
			47	AUC25
48	49			CA*FPD
			50	CA*CD90
			51	CA*CD75
			52	CA*CD50
			53	CA*CD25
54	58			FPD*CD90
55	59			FPD*CD75
56	60			FPD*CD50
57	61			FPD*CD25
			62 to 66 / 38 to 42	K
			67 to 99 / 43 to 157	Wavelets

X: Specific to TdP risk study. X: Specific to channel study.

Table D. Indices and names of the dictionary entries.

drug	Na IC50 (nM)	Ca IC50 (nM)	K IC50 (nM)	EFTPC (nM)
Ajmaline	8200.0	71000.0	1040.0	1500.0
Amiodarone	4800.0	270.0	30.0	0.5
Amitriptyline	20000.0	11600.0	3280.0	41.0
Bepridil*	3700.0	211.0	33.0	33.0
Chlorpromazine*	4300.0	nan	1470.0	38.0
Cibenzoline	7800.0	30000.0	22600.0	976.0
Cisapride*	14700.0	nan	6.5	4.9
Desipramine	1520.0	1709.0	1390.0	108.0
Diltiazem*	9000.0	450.0	17300.0	122.0
Diphenhydramine	41000.0	228000.0	5200.0	34.0
Dofetilide*	300000.0	60000.0	5.0	2.0
Fluvoxamine	39400.0	4900.0	3100.0	377.0
Haloperidol	7000.0	1700.0	27.0	3.6
Imipramine	3600.0	8300.0	3400.0	106.0
Mexiletine*	43000.0	100000.0	50000.0	4129.0
Mibefradil	980.0	156.0	1800.0	12.0
Nifedipine*	37000.0	60.0	275000.0	7.7
Nitrendipine*	36000.0	0.35	10000.0	3.02
Phenytoin	49000.0	103000.0	100000.0	4500.0
Pimozide*	54.0	162.0	20.0	1.0
Prenylamine	2520.0	1240.0	65.0	17.0
Propafenone	1190.0	1800.0	440.0	241.0
Propranolol	2100.0	18000.0	2828.0	26.0
Quetiapine	16900.0	10400.0	5800.0	33.0
Quinidine*	16600.0	15600.0	300.0	924.0
Risperidone*	102000.0	73000.0	150.0	1.81
Sertindole	2300.0	8900.0	14.0	1.59
Tedisamil	20000.0	nan	2500.0	85.0
Terfenadine*	971.0	375.0	8.9	9.0
Thioridazine	1830.0	1300.0	33.0	208.0
Verapamil*	41500.0	100.0	143.0	81.0

Table E. Drugs known as torsadogenic (red) and non-torsadogenic (green) with their IC50 and EFTPC from Mirams *et al.* *: CiPA compound [1].

drug	Na IC50 (nM)	Ca IC50 (nM)	K IC50 (nM)	EFTPC (nM)
Amiodarone	15900.0	1900.0	860.0	0.8
Astemizole*	3000.0	1100.0	4.0	0.3
Bepridil*	2300.0	1000.0	160.0	35.0
Ceftriaxone	555900.0	153800.0	445700.0	23170.0
Chlorpromazine*	3000.0	3400.0	1500.0	38.0
Cilostazol	93700.0	91200.0	13800.0	128.0
Cisapride*	337000.0	11800.0	20.0	3.0
Clozapine*	15100.0	3600.0	2300.0	71.0
Dasatinib	76300.0	81100.0	24500.0	41.0
Diazepam	306400.0	30500.0	53200.0	29.0
Diltiazem*	22400.0	760.0	13200.0	122.0
Disopyramide*	168400.0	1036700.0	14400.0	742.0
Dofetilide*	162100.0	26700.0	30.0	2.0
Donepezil	38500.0	34300.0	700.0	3.0
Droperidol*	22700.0	7600.0	60.0	16.0
Duloxetine	5100.0	2800.0	3800.0	16.0
Flecainide	6200.0	27100.0	1500.0	753.0
Halofantrine	331200.0	1900.0	380.0	172.0
Haloperidol	4300.0	1300.0	40.0	4.0
Ibutilide*	42500.0	62500.0	18.0	140.0
Lamivudine	1571400.0	54200.0	2054000.0	19540.0
Linezolid	2644500.0	105400.0	1147200.0	59110.0
Loratadine*	28900.0	11400.0	6100.0	0.4
Methadone	31800.0	37400.0	3500.0	507.0
Metronidazole	2073200.0	177900.0	1340200.0	187000.0
Mibefradil	5600.0	510.0	1700.0	12.0
Mitoxantrone	93500.0	22500.0	539400.0	225.0
Moxifloxacin	1112000.0	173000.0	86200.0	10960.0
Nifedipine*	88500.0	12.0	44000.0	8.0
Nilotinib	13300.0	17500.0	1000.0	172.0
Nitrendipine*	21600.0	25.0	24600.0	3.0
Paliperidone	109000.0	193900.0	780.0	69.0
Paroxetine	9800.0	3900.0	1900.0	14.0
Pentobarbital	2686000.0	299000.0	1433900.0	5171.0
Phenytoin	72400.0	21900.0	147000.0	4360.0
Pimozide*	1100.0	240.0	40.0	0.5
Piperacillin	2433800.0	1226000.0	3405100.0	1378000.0
Procainamide	746600.0	389500.0	272400.0	54180.0
Quinidine*	14600.0	6400.0	720.0	3237.0
Raltegravir	824200.0	246700.0	782800.0	7000.0
Ribavirin	2997500.0	622500.0	967000.0	27880.0
Risperidone*	43400.0	34200.0	260.0	2.0
Saquinavir	12100.0	1900.0	16900.0	130.0
Sertindole	6900.0	6300.0	33.0	2.0
Sitagliptin	1220800.0	147100.0	174700.0	442.0
Solifenacin	1500.0	4300.0	280.0	3.0
Sotalol*	7013900.0	193300.0	111400.0	14690.0
Sparfloxacin	2555000.0	88800.0	22100.0	1766.0
Sunitinib	16500.0	33400.0	1200.0	13.0
Telbivudine	1095200.0	713900.0	422700.0	19720.0
Terfenadine*	2000.0	930.0	50.0	9.0
Terodiline	7400.0	4800.0	650.0	145.0
Thioridazine	1400.0	3500.0	500.0	980.0
Verapamil*	32500.0	200.0	250.0	88.0
Voriconazole	1550500.0	414200.0	490900.0	7563.0

Table F. Drugs known as torsadogenic (red) and non-torsadogenic (green) with their IC50 and EFTPC from Kramer *et al.* *: CiPA compound [1].

Fig A. Confusion matrices obtained for TdP risk classification. From top to bottom we have respectively 1 component in $\mathbb{R}$, 2 components in $\mathbb{R}$, 3 components in $\mathbb{R}$, 3 components in $\mathbb{R}$ and 1 component in $\mathbb{R}^2$, 3 components in $\mathbb{R}$ and 2 components in $\mathbb{R}^2$ and 3 components in $\mathbb{R}$ and 3 components in $\mathbb{R}^2$. The left column corresponds to the training set and the right column to the validation set. No: No TdP risk. Yes: TdP risk.

Compound	Concentration 1			Concentration 2			Concentration 3			Concentration 4		
	hERG	Cav1.2	Nav1.5	hERG	Cav1.2	Nav1.5	hERG	Cav1.2	Nav1.5	hERG	Cav1.2	Nav1.5
Loratadine	100	100	100	100	100	100	99.8	99.9	100	99.5	99.7	99.9
Ibutilide	99.4	100	100	94.7	100	100	64.3	100	100	15.3	99.8	99.8
Droperidol	65.4	99.6	99.9	37.5	98.7	99.6	15.9	96.0	98.6	5.7	88.4	95.8
Mexiletine	99.8	99.9	99.7	98.4	99.2	97.4	86.1	92.6	79.2	38.3	55.6	27.5
Dofetilide	99.0	100	100	96.8	100	100	90.4	100	100	75.0	100	100
Diltiazem	99.9	98.7	100	99.2	88.4	99.6	93.0	43.2	95.7	56.9	7.1	69.1
Chlorpromazine	94.0	97.3	96.9	83.3	91.9	90.9	61.2	78.2	76.0	33.3	53.1	50.0
Clozapine	96.0	97.4	99.4	88.4	92.3	98.0	70.8	79.1	94.1	43.4	54.5	83.4
Clarithromycine	99.7	NA	NA	97.1	NA	NA	76.7	NA	NA	24.8	NA	NA
Cisapride	86.2	100	100	66.7	99.9	100	38.8	99.7	100	16.7	99.2	100
Bepridil	94.1	99.0	99.6	61.5	90.9	95.8	13.8	50.0	69.7	1.6	9.1	18.7
Azimilide	NA	NA	NA	NA	NA	NA	NA	NA	NA	NA	NA	NA

Table G. Percentage of activity using a Hill coefficient equals to 1.

Stop criterion based on the success rate variation

Starting from the cost function defined in 12, let's compute the case where all the samples are well classified but with the lowest probability ($\widehat{l_i} = l_i$ and $\widehat{p_i} = \frac{1}{2}, \forall i$):

$$s_{N_s} = -\frac{1}{N_s}\sum_{i=1}^{N_s}\widehat{p_i}\Bigg\{\sum_{j=1}^{k}\Big[\frac{N_s}{n_j}\delta_j(\widehat{l_i})\delta_j(l_i) - \frac{N_s}{n_j}\alpha_j\sum_{\substack{m=1\\m\neq j}}^{k}\delta_m(\widehat{l_i})\delta_j(l_i)\Big]\Bigg\}. \tag{15}$$

As all samples are well classified with a probability equals to $\frac{1}{2}$, we have:

$$\widetilde{s}_{N_s} = -\frac{1}{N_s}\sum_{i=1}^{N_s}\frac{1}{2}\sum_{j=1}^{k}\delta_j(\widehat{l_i})\delta_j(l_i). \tag{16}$$

We know how many samples are in each class. Then,

$$\widetilde{s}_{N_s} = -\frac{1}{N_s}\Big(\frac{1}{2}\frac{N_s n_1}{n_1} + ... + \frac{1}{2}\frac{N_s n_k}{n_k}\Big). \tag{17}$$

$$\Longrightarrow \widetilde{s}_{N_s} = -\frac{k}{2}. \tag{18}$$

If now, just 1 sample x is wrongly classified, we have,

$$s_{N_s} = -\frac{1}{N_s}\Big(\frac{1}{2}\frac{N_s n_1}{n_1} + ... + \frac{1}{2}\frac{N_s n_{j-1}}{n_{j-1}} + \frac{1}{2}\frac{N_s(n_j - 1)}{n_j} + \frac{1}{2}\frac{N_s n_{j+1}}{n_{j+1}} + ... + \frac{1}{2}\frac{N_s n_k}{n_k} - \widehat{p_x}\alpha_j\frac{N_s}{n_j}\Big). \tag{19}$$

$$\Longrightarrow s_{N_s} = -\frac{k}{2} + \frac{1 + 2\widehat{p_x}\alpha_j}{2n_j}. \tag{20}$$

Then, the variation between the two costs is:

$$\Delta = \mid \widetilde{s}_{N_s} - s_{N_s} \mid. \tag{21}$$

$$\Longrightarrow \Delta = \frac{1 + 2\widehat{p_x}\alpha_j}{2n_j}. \tag{22}$$

The minimum variation which ensures that the classification did not change is described in Equation 23.

$$\Delta_{min} = \min_{j,\widehat{p_x}}\Big(\frac{1 + 2\widehat{p_x}\alpha_j}{2n_j}\Big). \tag{23}$$

Regardless of the class, the minimum is obtained for $\widehat{p_x} = \frac{1}{2}$. Hence,

$$\Delta_{min} = \min_{j}\Big(\frac{1 + \alpha_j}{2n_j}\Big). \tag{24}$$

A criterion to add a new dimension could be:

$$\mid s_{N_s}^{new} - s_{N_s}^{old} \mid < \alpha\Delta_{min}. \tag{25}$$

With $\alpha \in [0, 1]$, a user parameter. If $\alpha = 1$, we are sure that the classification did not change. If $\alpha < 1$, the classification will be the same, but margins will still increase.

As an example, in the TdP classification we have two classes. If we train on the first 76 compounds, 50 of them have a TdP risk and 36 of them do not have a TdP risk. In

the case we want to penalize the false negative rate (drug labeled as no TdP risk instead of TdP risk) with $\alpha = 2$, we finally obtain:

$$(\alpha_j, n_j) = \begin{cases} (1, 50) \text{ if } j = \text{ TdP risk} \\ (2, 36) \text{ if } j = \text{ no TdP risk} \end{cases} . \quad (26)$$

Then, we have $\Delta_{min} = 0.02$. It means that if the difference between the old cost and the new cost is under than 0.02, we are sure that the classification is the same and only probabilities changed.

Concerning the cross-validation part, we made the choice to repeat the k-fold 500 times. B Fig shows us how the cost is impacted by the number of k-fold. We can see that the cost is more stable for a low number of components. Moreover, the cost is under the Δ_{min} threshold for most of the number of k-fold. Under this condition, the number of repeated k-fold has less importance.

Fig B. Impact of the number of repeated k-fold on the cost function in the torsadogenicity classification with the $\mathbf{\Delta_{min} = 0.02}$ range.

References

1. Colatsky T, Fermini B, Gintant G, Pierson JB, Sager P, Sekino Y, et al. The comprehensive in vitro proarrhythmia assay (CiPA) initiative-Update on progress. Journal of pharmacological and toxicological methods. 2016;81:15–20.
